# Supplementary material for: CDK4 phosphorylation status and rational use for combining CDK4/6 and BRAF/MEK inhibition in advanced thyroid carcinomas
Source: Front Endocrinol (Lausanne). 2023 Oct 26;14:1247542. doi: 10.3389/fendo.2023.1247542 (PMC10641312; doi:10.3389/fendo.2023.1247542)
Supplement: Supplementary file 4 [file Presentation_2.pdf]

Supplementary Figure S4

Immunohistochemical staining for Hematoxylin/Eosin (HE), KI67 and p16 performed in serial sectioning of FFPE samples from ATC, PDTC, PTC and normal thyroid tissues

ATC4 - JPI21 - pCDK4 profile A

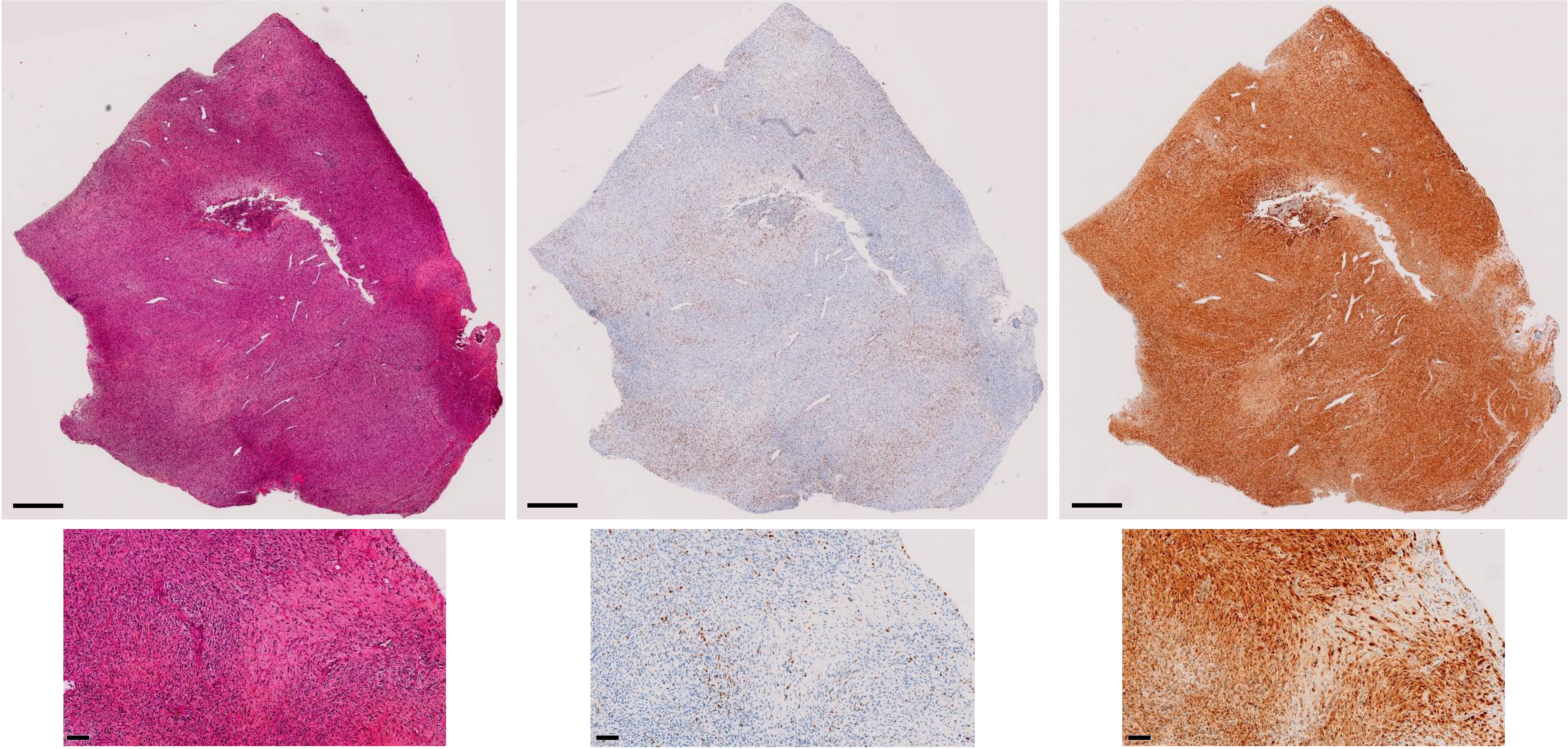

HE KI67 p16

Scale bar = 1mm
Scale bar = 100µm

ATC7 - JPI74 - pCDK4 profile A

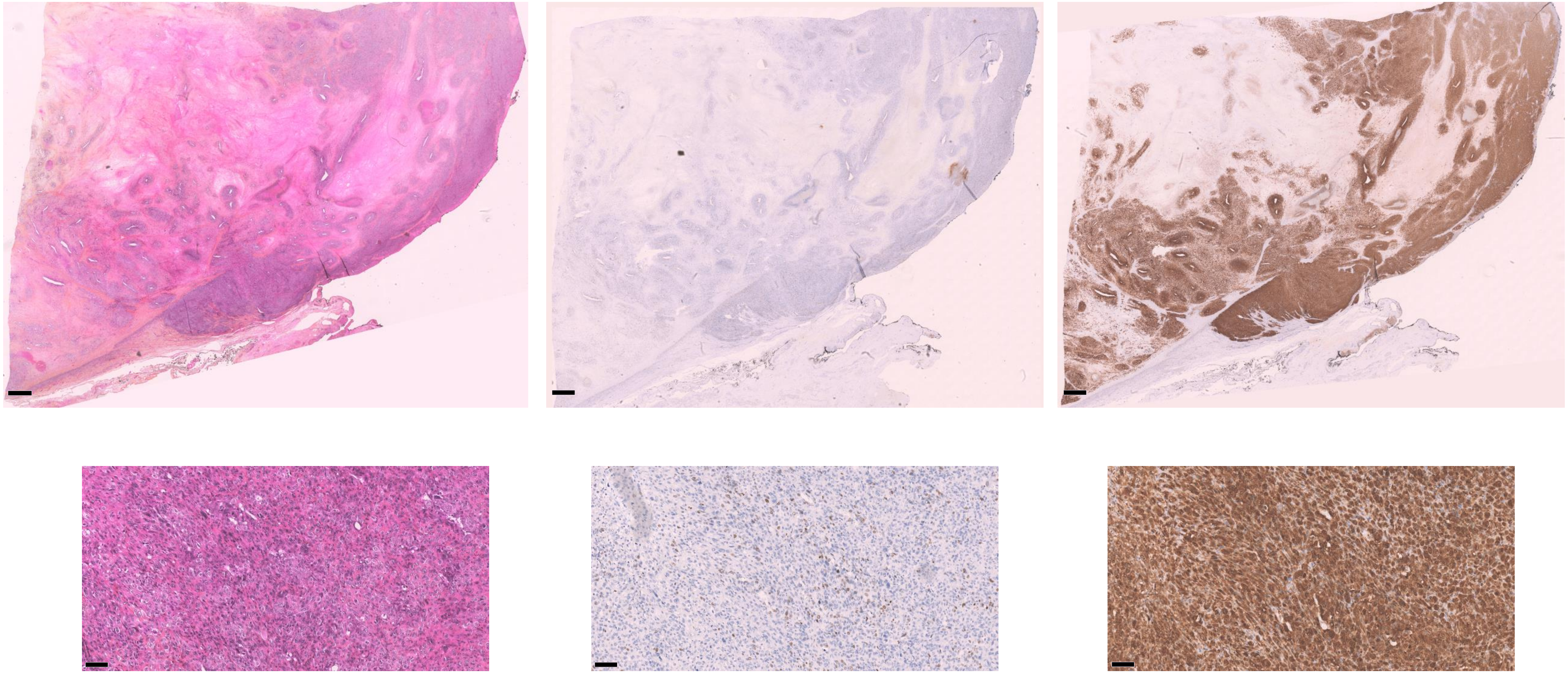

HE

KI67

p16

Scale bar = 1mm

Scale bar = 100µm

ATC1 - pCDK4 profile A

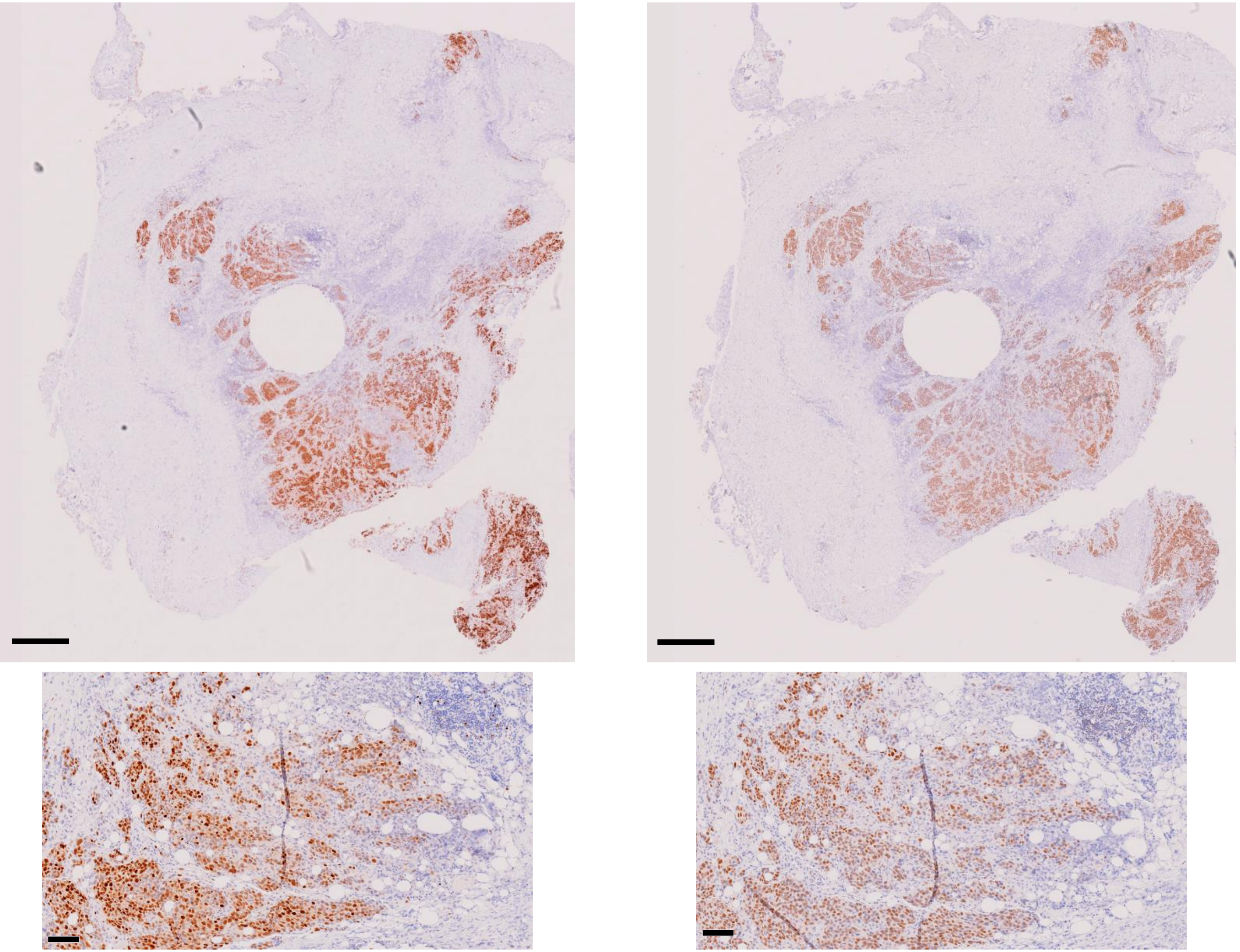

KI67 **p16**

Staining done with different set of antibodies (as detailed in Table S2)

Scale bar = 1mm

Scale bar = 100µm

ATC2 - pCDK4 profile A

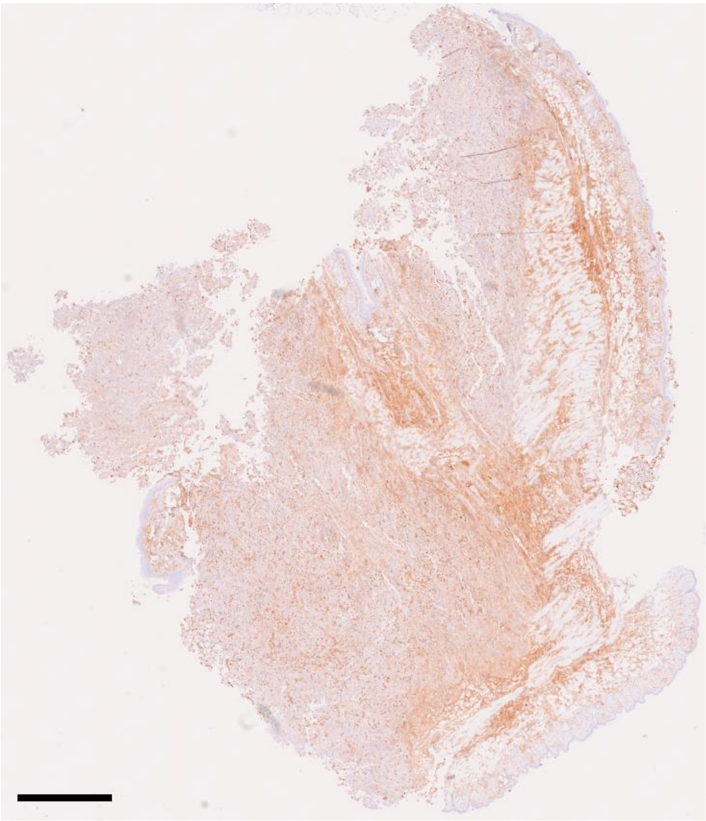

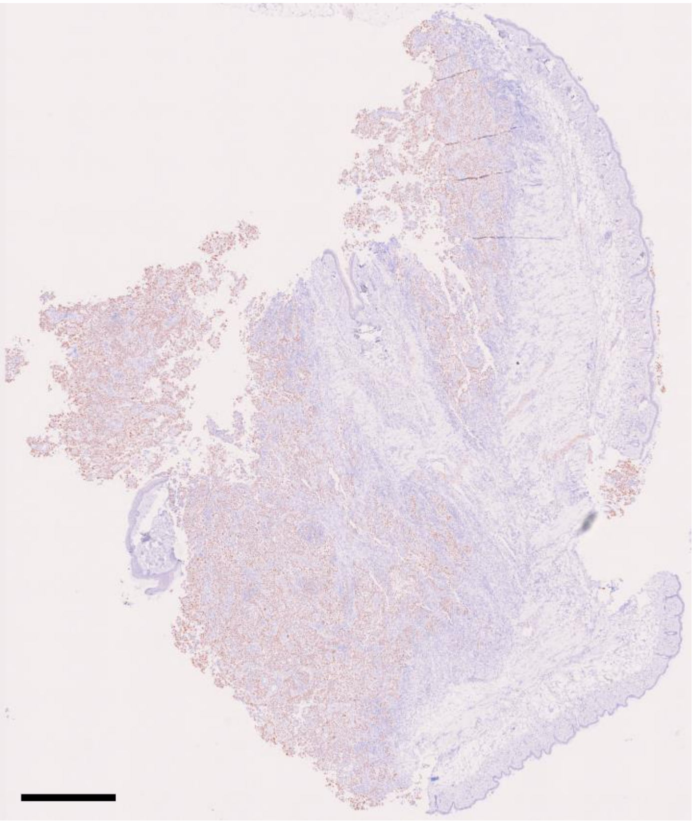

Staining done with different set of antibodies (as detailed in Table S2)

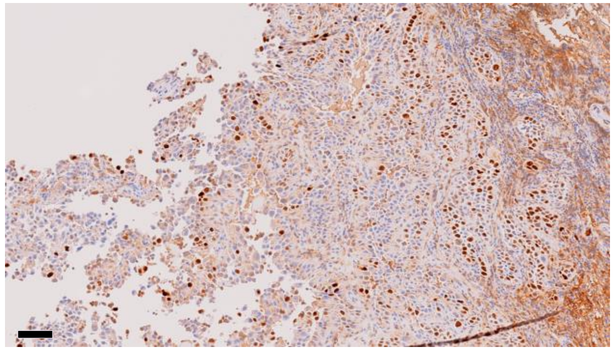

KI67

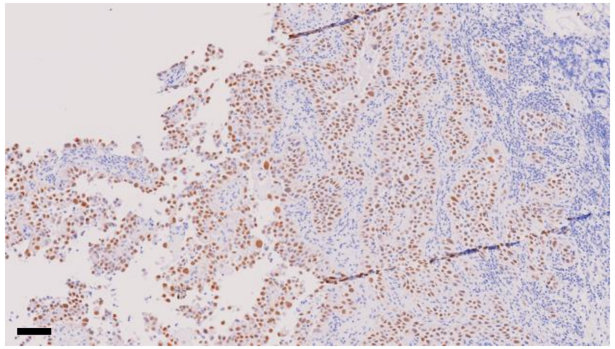

p16

Scale bar = 1mm
Scale bar = 100µm

ATC3 - pCDK4 profile A

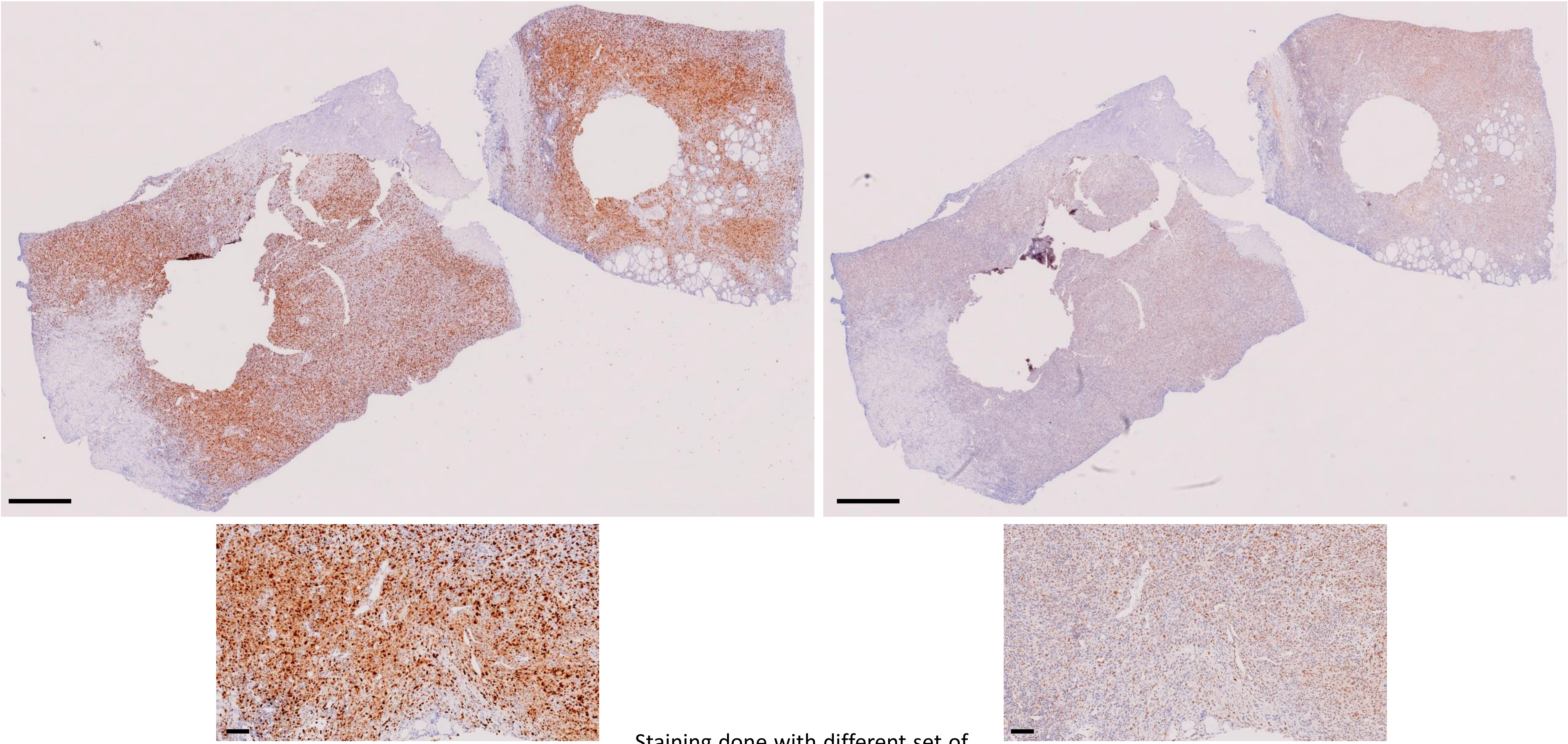

KI67

Staining done with different set of antibodies (as detailed in Table S2)

p16

Scale bar = 1mm

Scale bar = 100µm

ATC12 - JPI84 - pCDK4 profile L*

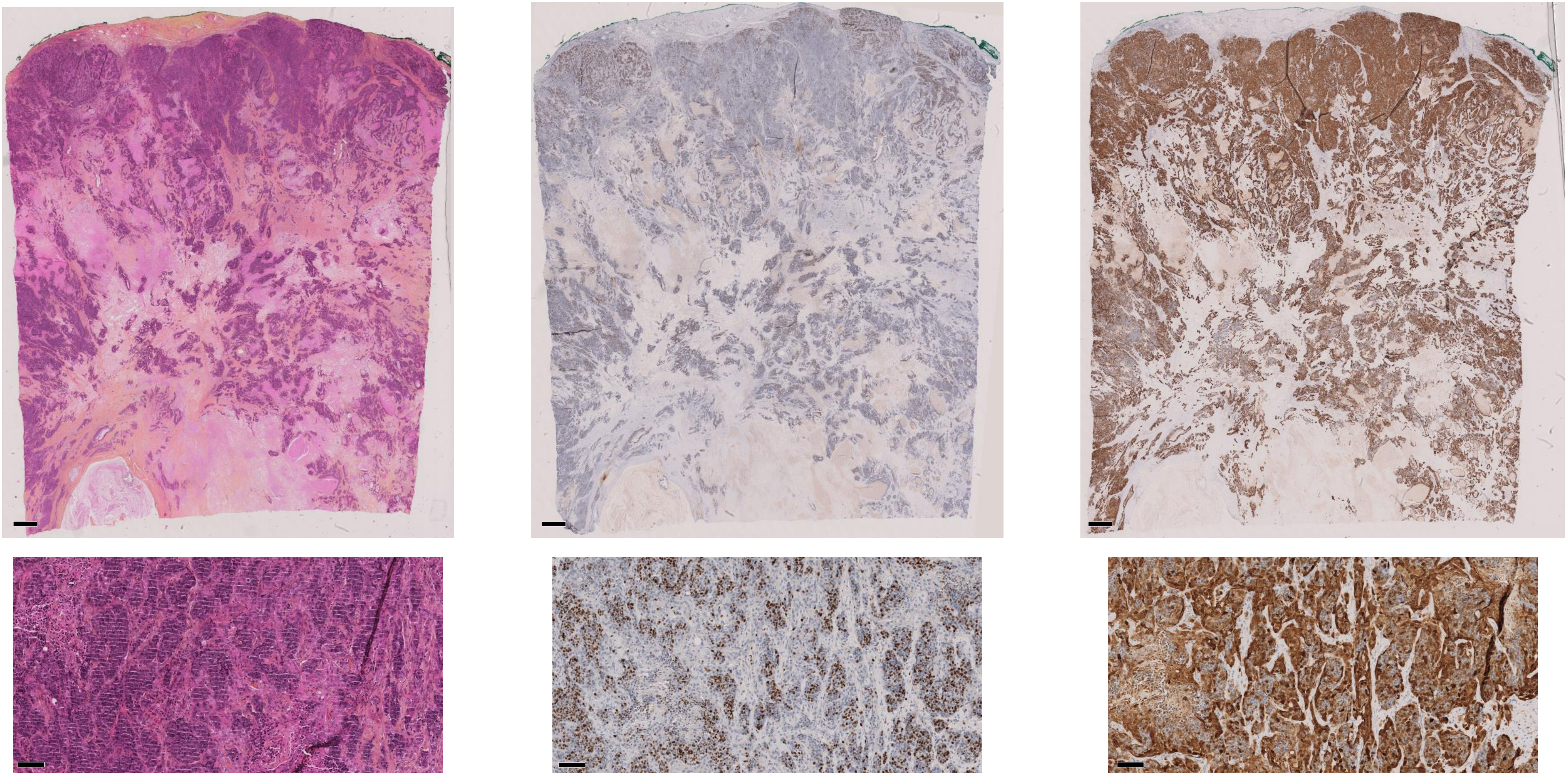

HE **KI67** **p16**

Scale bar = 1mm

Scale bar = 100µm

ATC22 - JPI37 - pCDK4 profile H

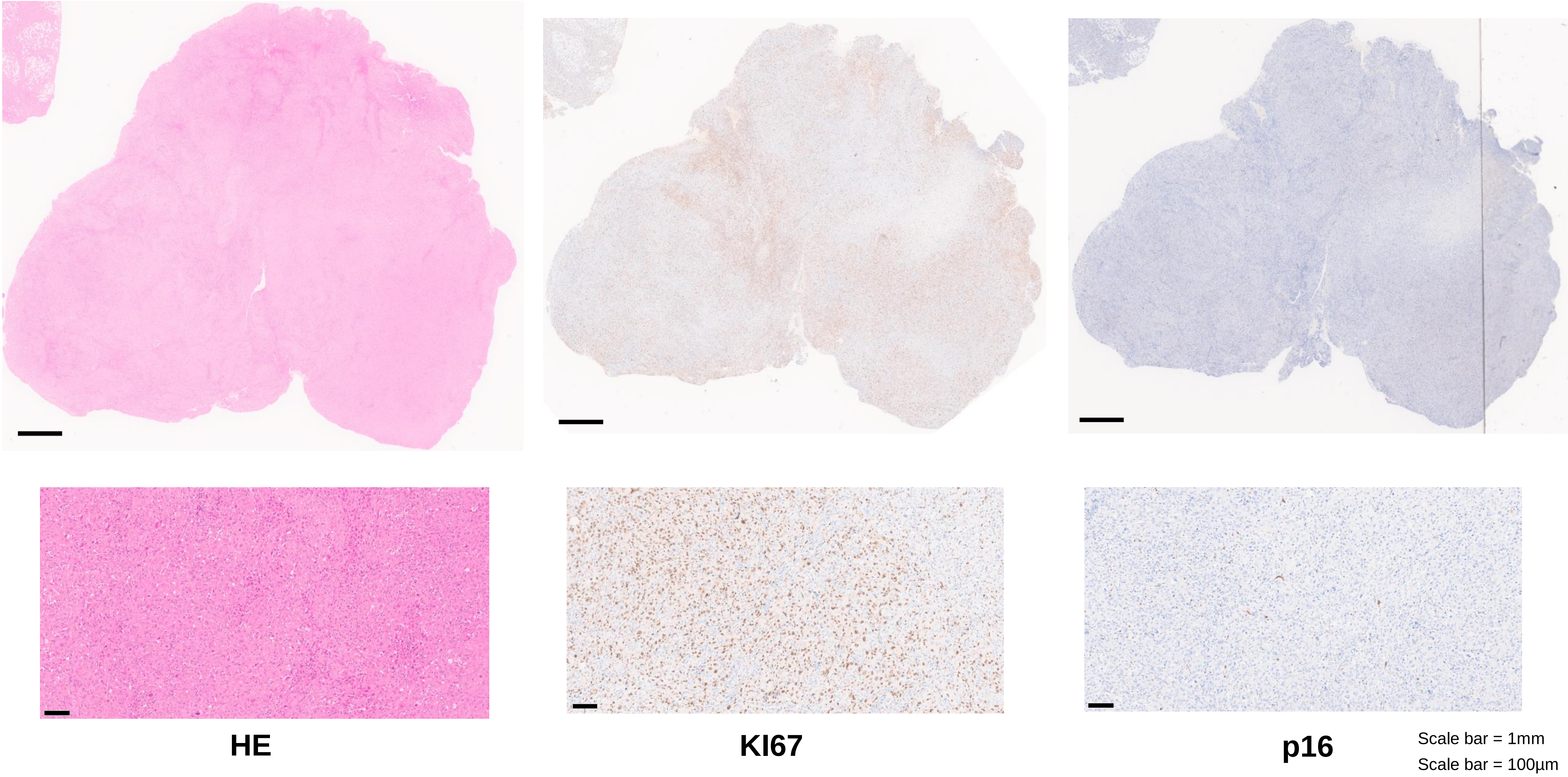

ATC21 - JPI41 - pCDK4 profile H

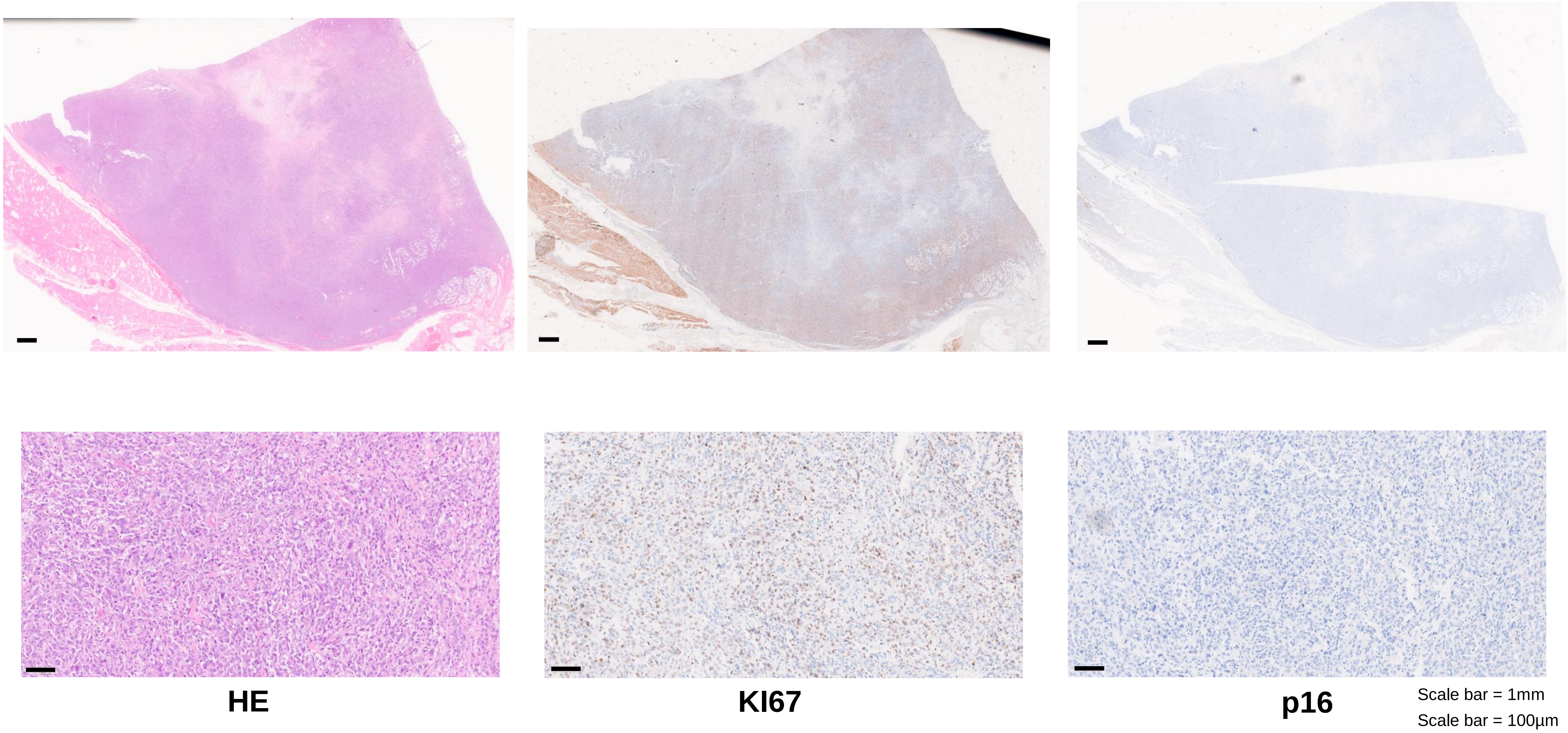

ATC18 - JPI66 - pCDK4 profile L

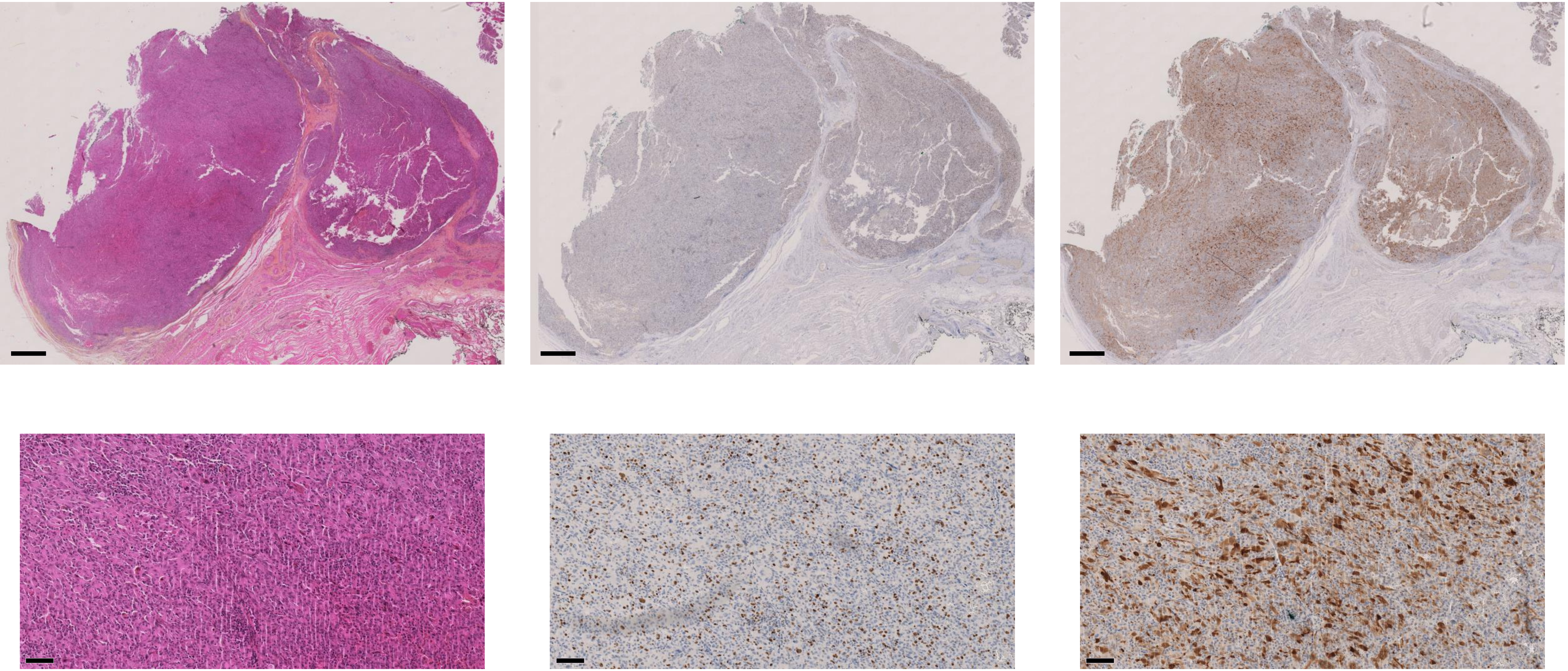

HE KI67 p16

Scale bar = 1mm
Scale bar = 100µm

ATC13 - JPI72 - pCDK4 profile L

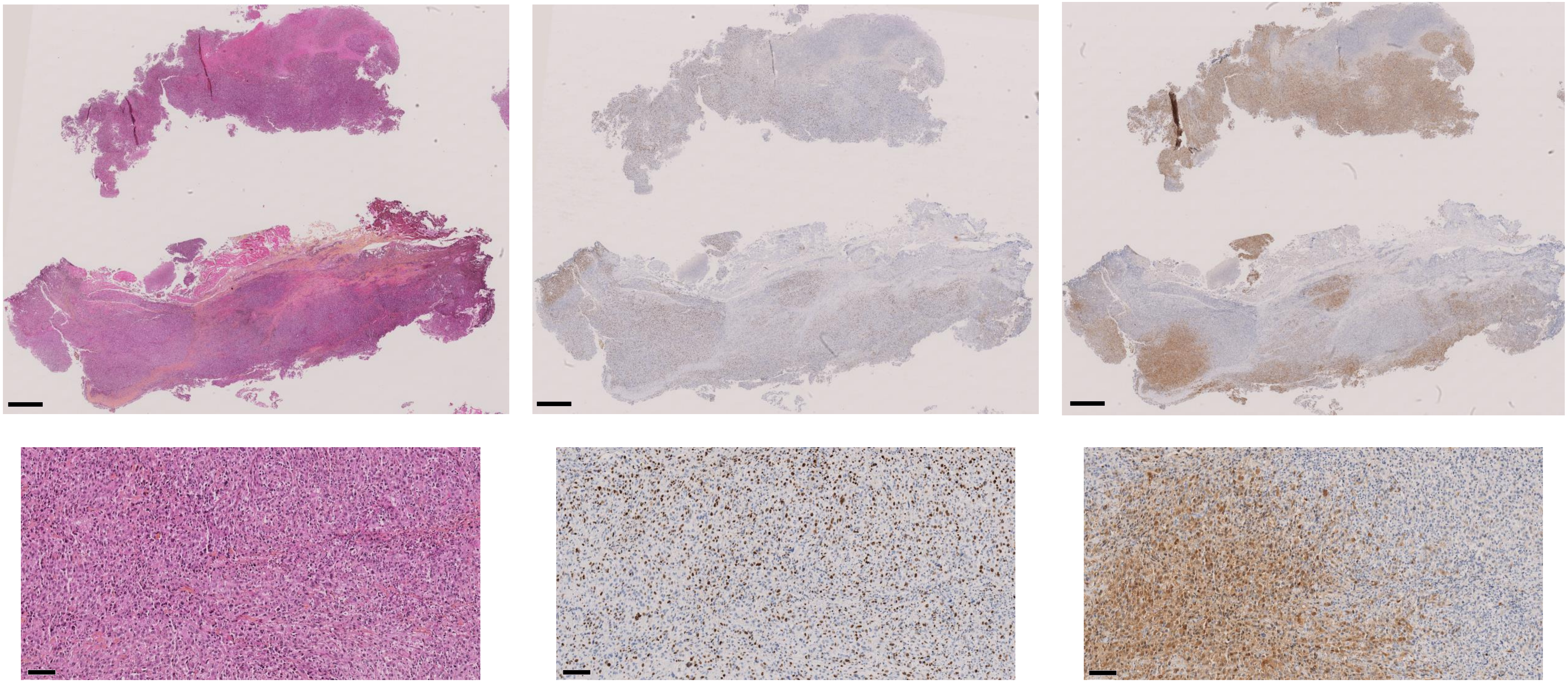

HE **KI67** **p16**

Scale bar = 1mm
Scale bar = 100µm

ATC14 - JPI26 - pCDK4 profile L

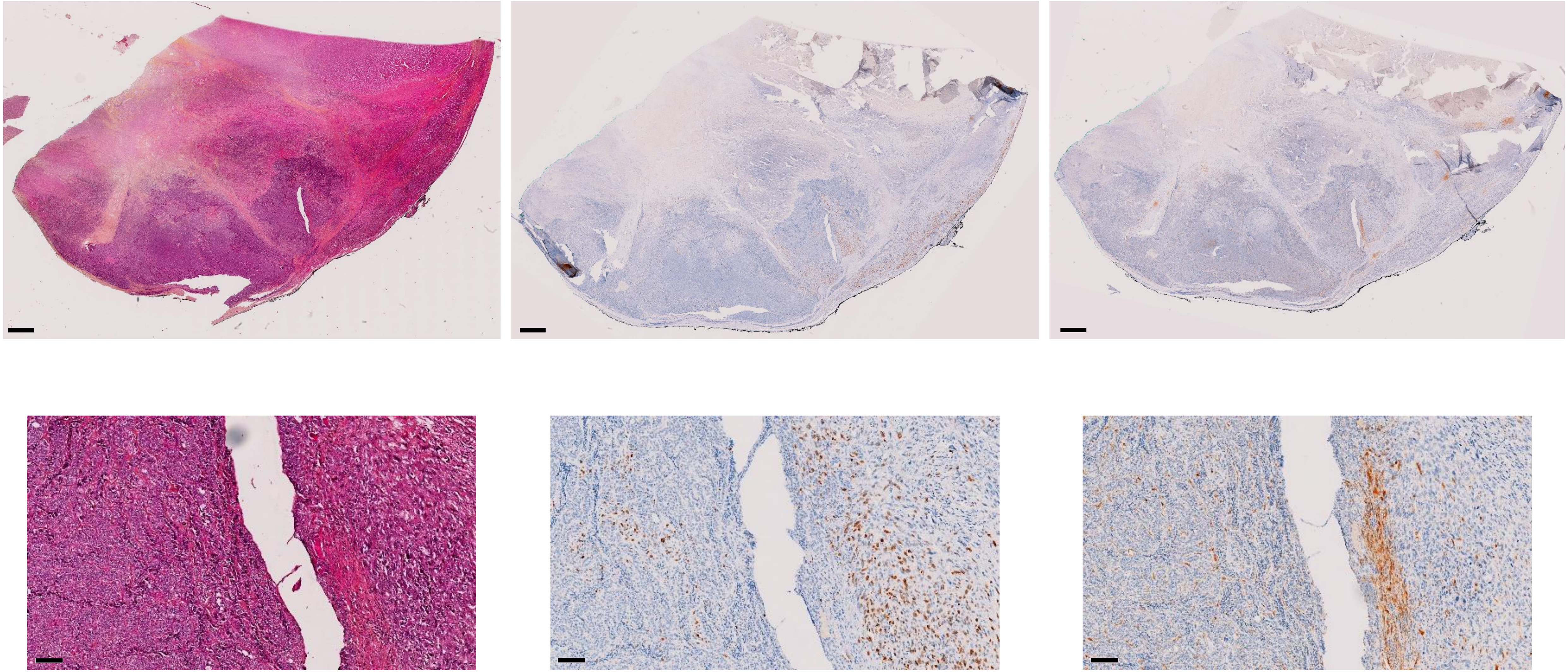

HE KI67 p16

Scale bar = 1mm
Scale bar = 100µm

ATC11 - JPI81 - pCDK4 profile L

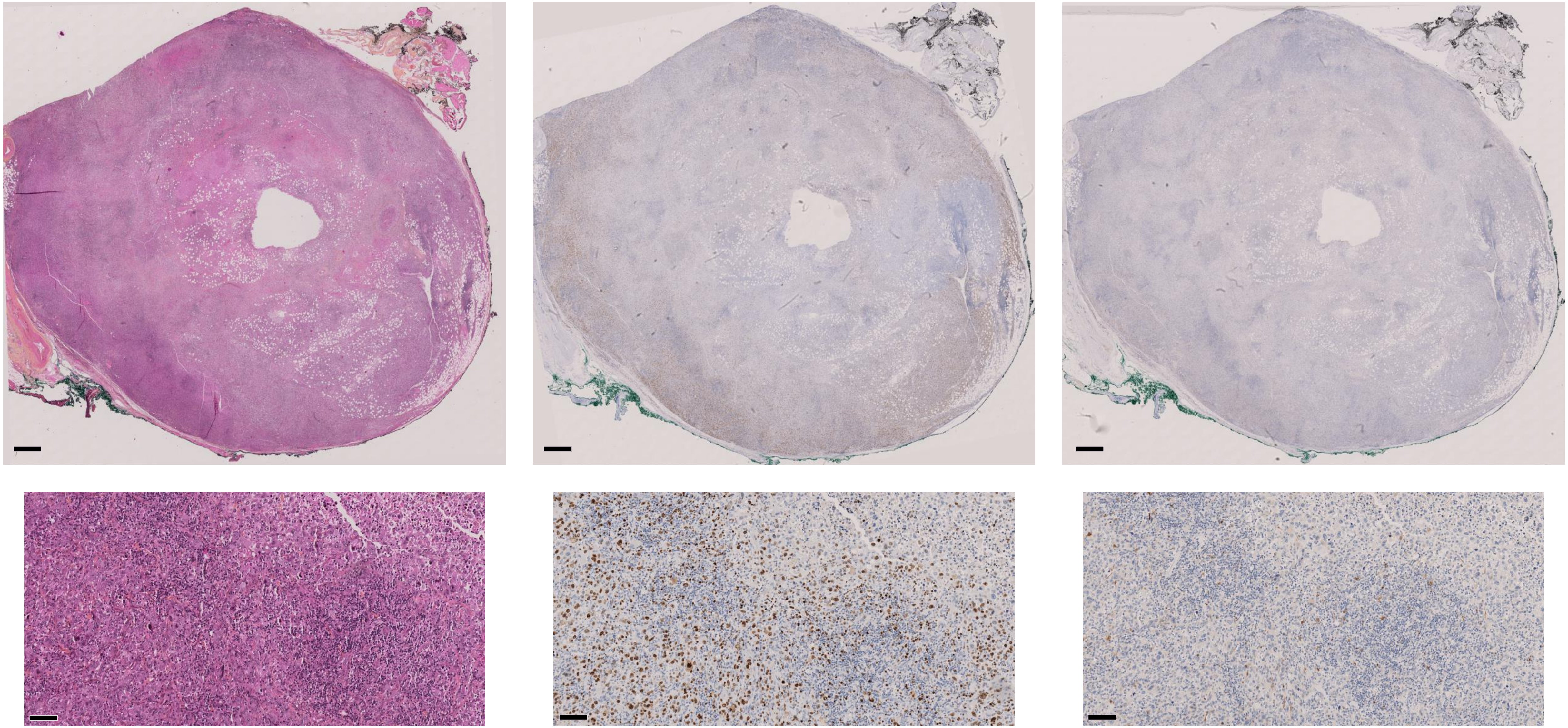

HE **KI67** **p16**

Scale bar = 1mm
Scale bar = 100µm

ATC19 - JPI76 - pCDK4 profile L

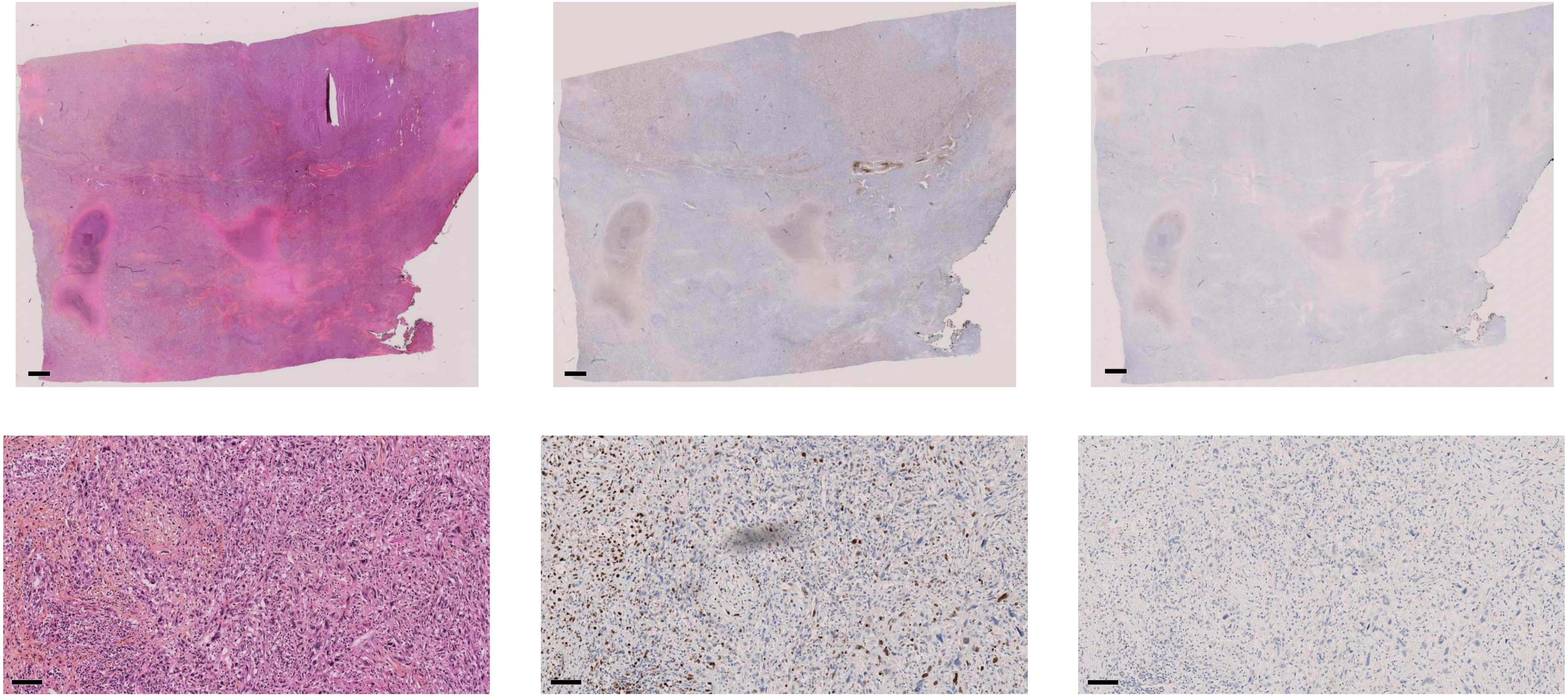

HE KI67 p16

Scale bar = 1mm
Scale bar = 100µm

PDTC1 - JPI25 - pCDK4 profile A

Vascular/lymphatic invasion of the tumor (TTF-1^{+} and PAX8^{+}) with lymphangitis carcinomatosa aspect

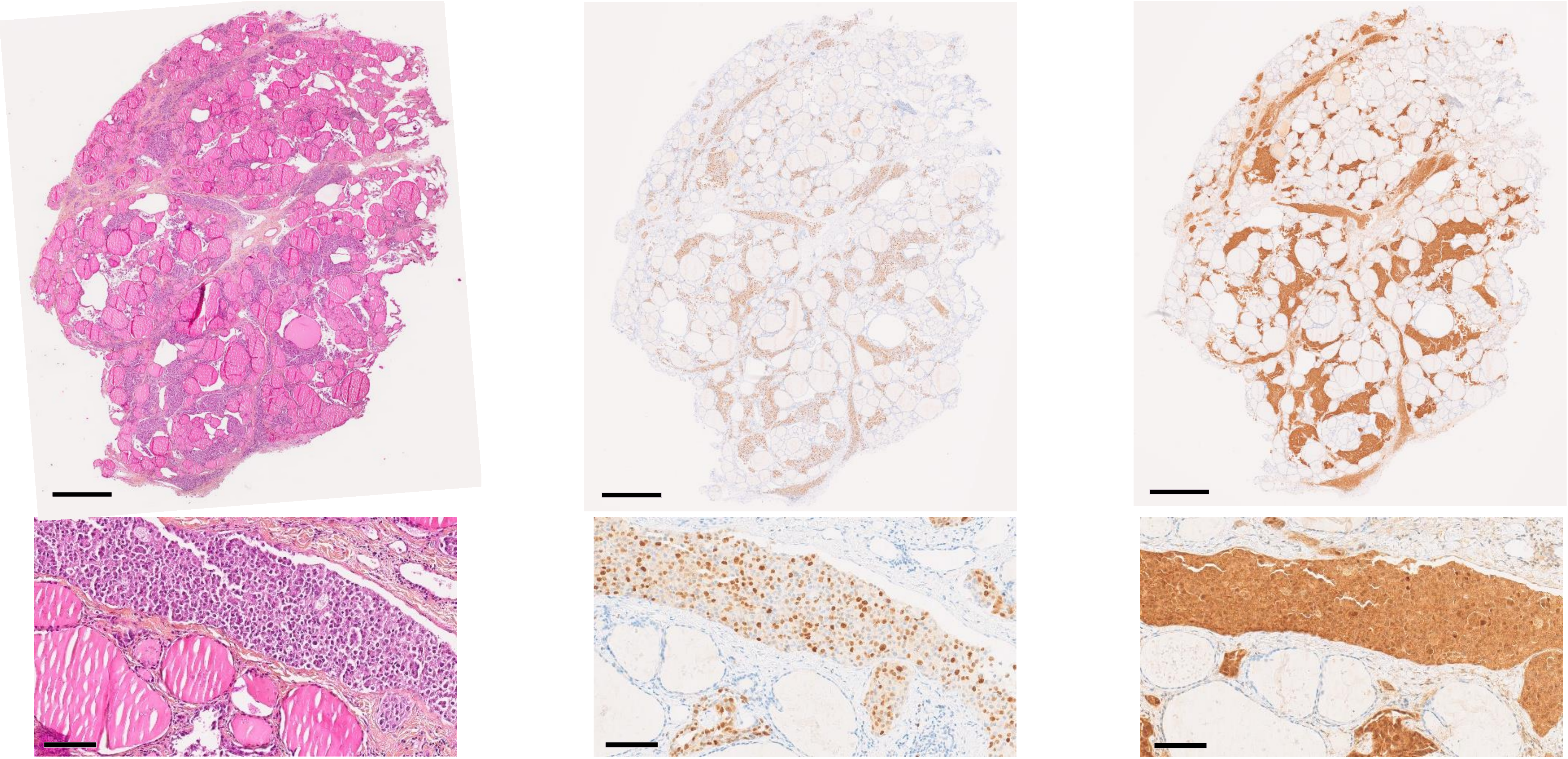

HE

KI67

p16

Scale bar = 1mm

Scale bar = 100µm

PDTC13 - JPI67 - pCDK4 profile H

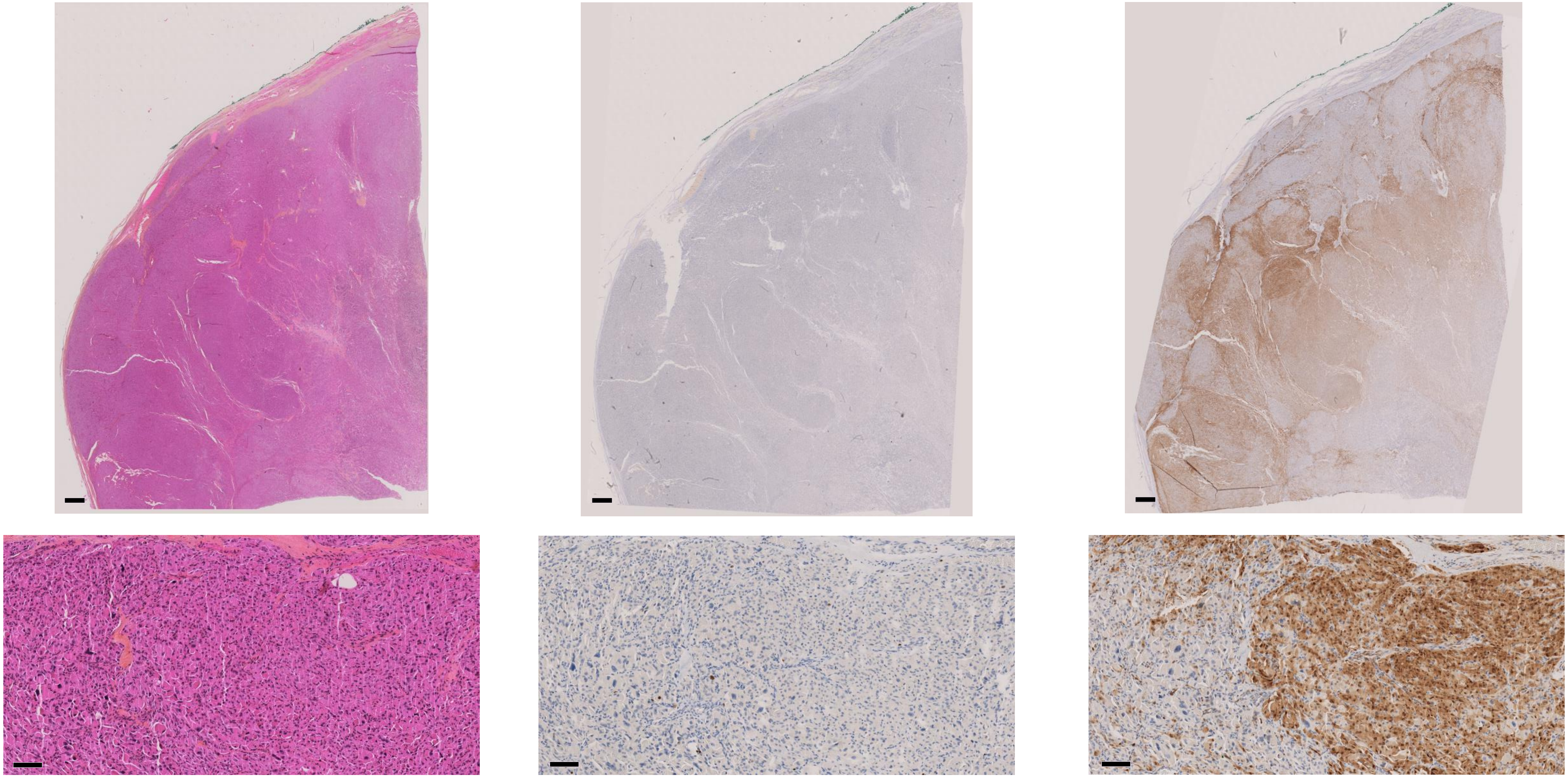

HE KI67 p16

Scale bar = 1mm
Scale bar = 100µm

PDTC20 - JPI29 - pCDK4 profile H

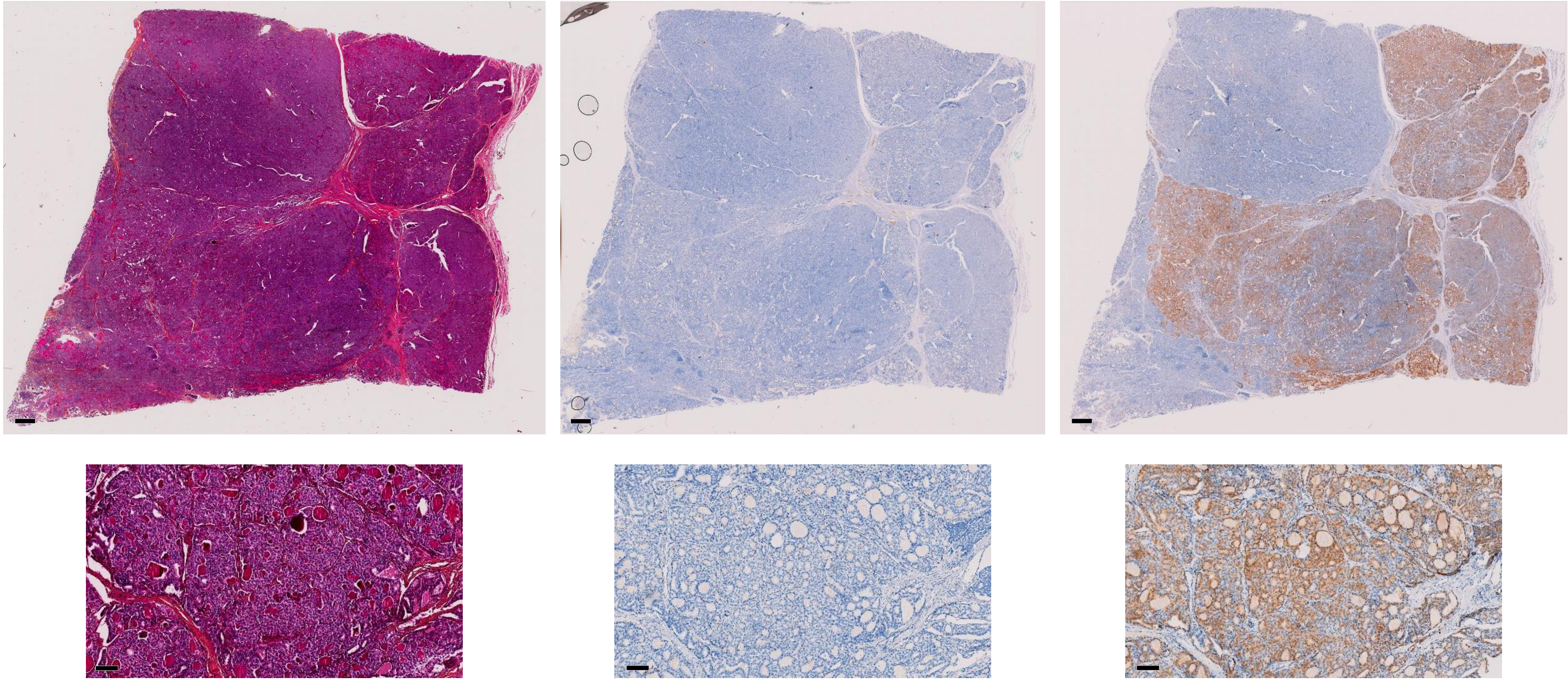

HE KI67 p16

Scale bar = 1mm

Scale bar = 100µm

PDTC14 - JPI75 - pCDK4 profile H

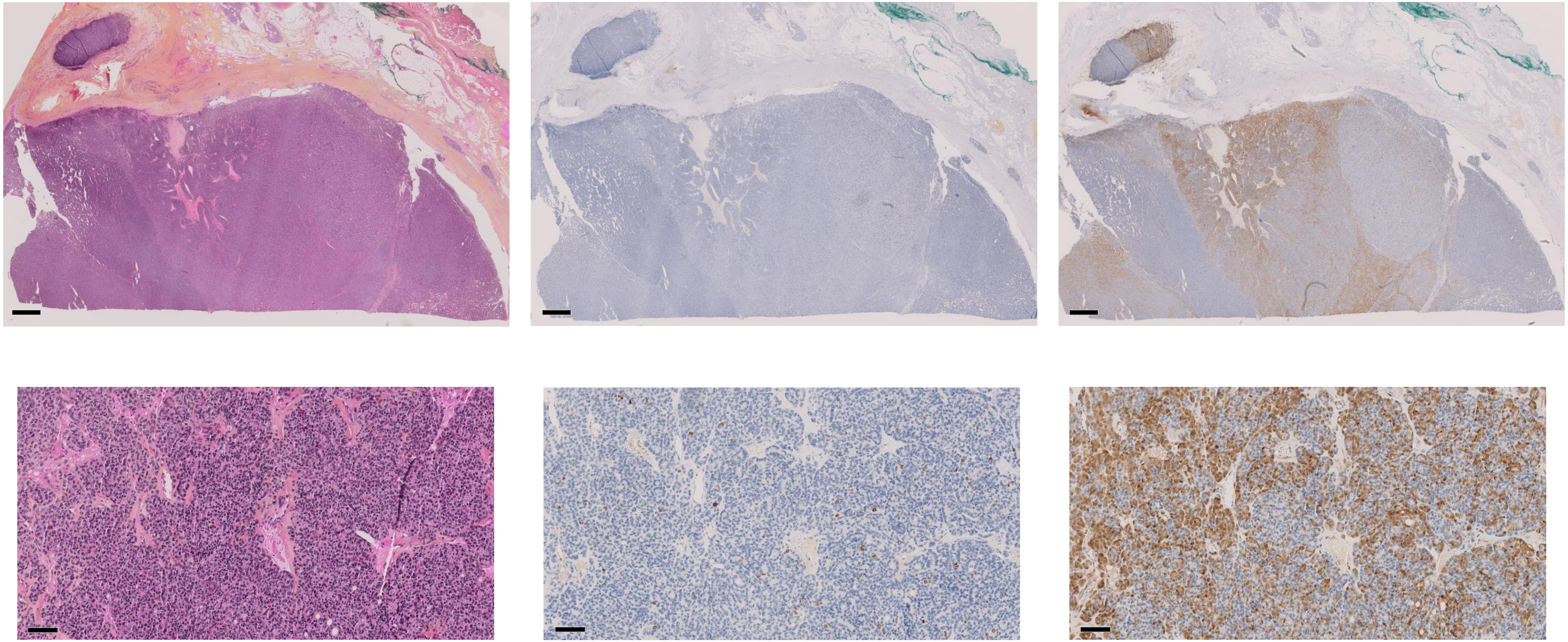

HE **KI67** **p16**

Scale bar = 1mm
Scale bar = 100µm

PDTC18 - JPI34 - pCDK4 profile H

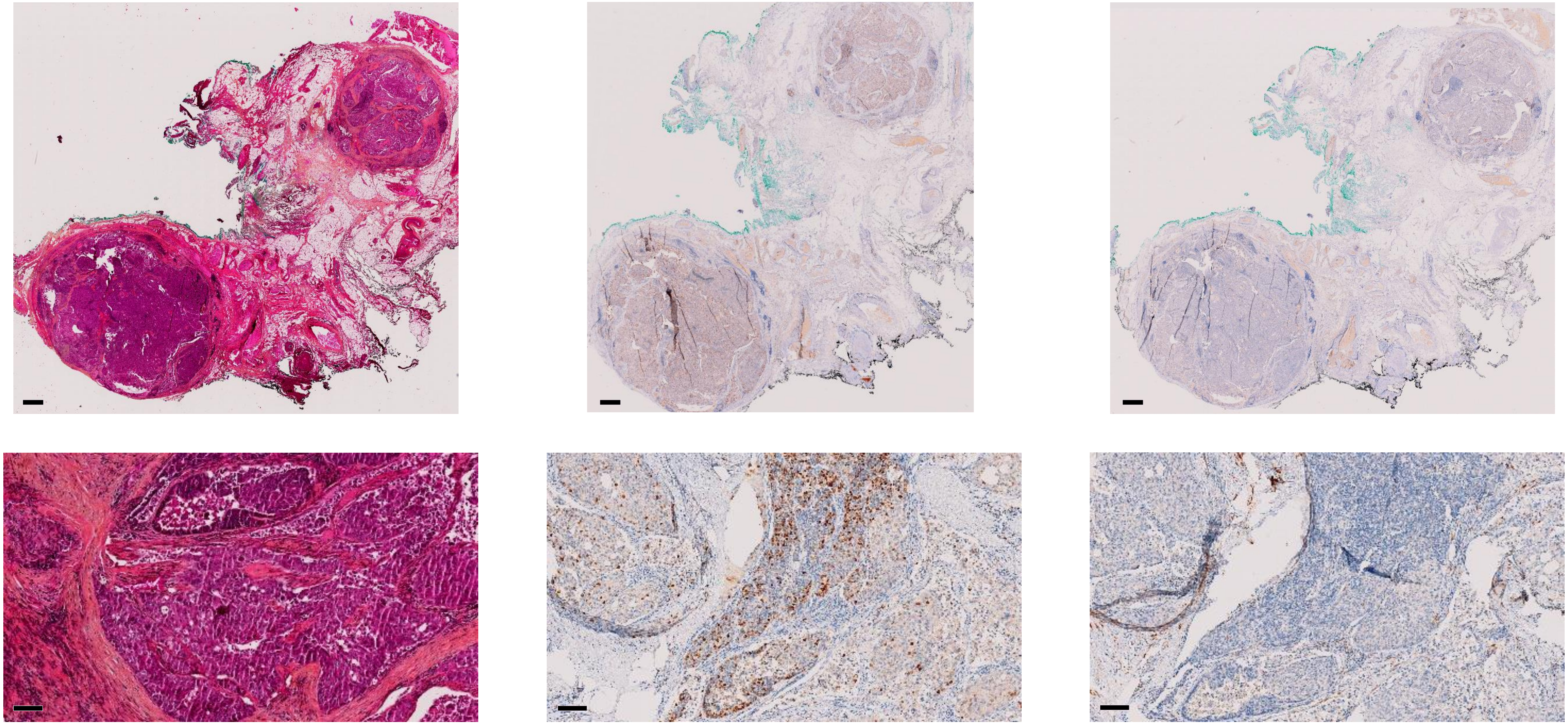

HE KI67 p16

Scale bar = 1mm
Scale bar = 100µm

PDTC12 - JPI19 - pCDK4 profile H

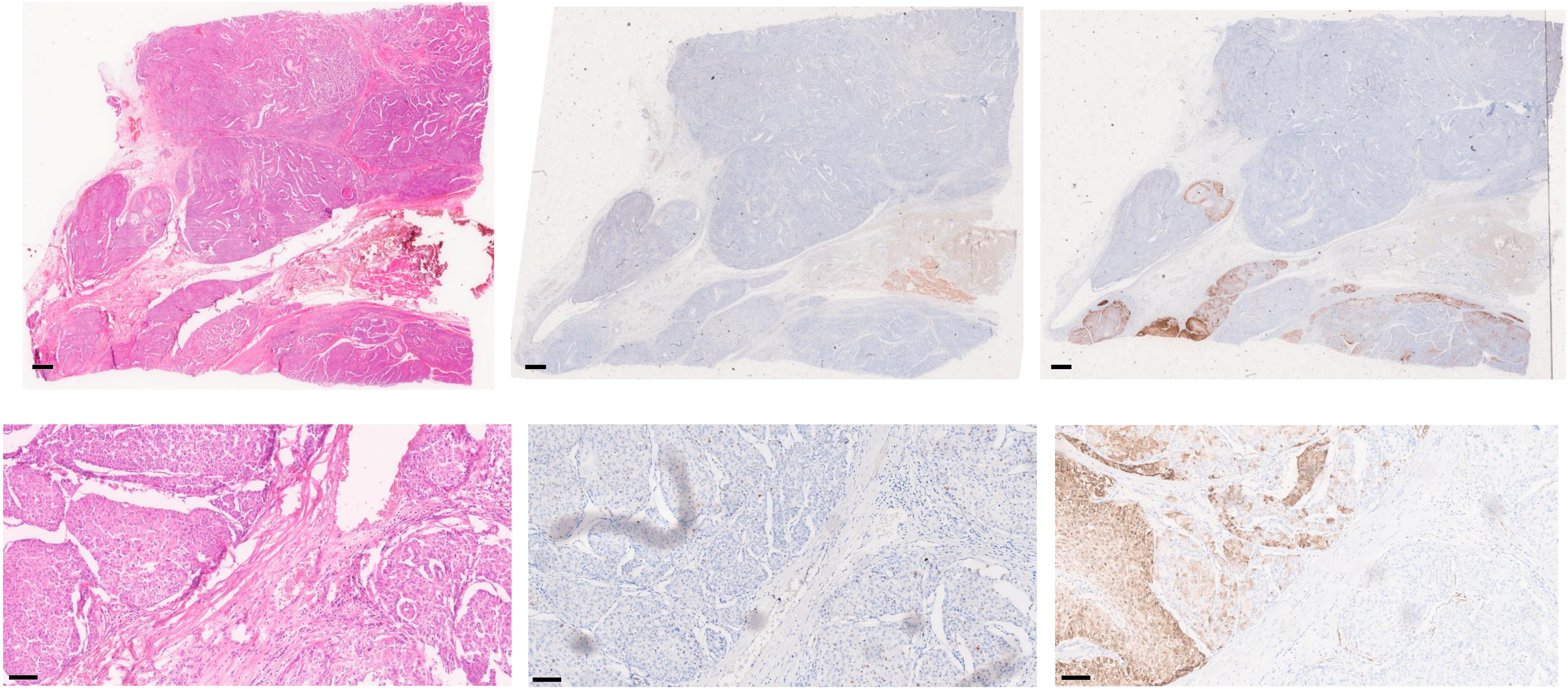

HE KI67 p16

Scale bar = 1mm
Scale bar = 100µm

PDTC16 - JPI73 - pCDK4 profile H

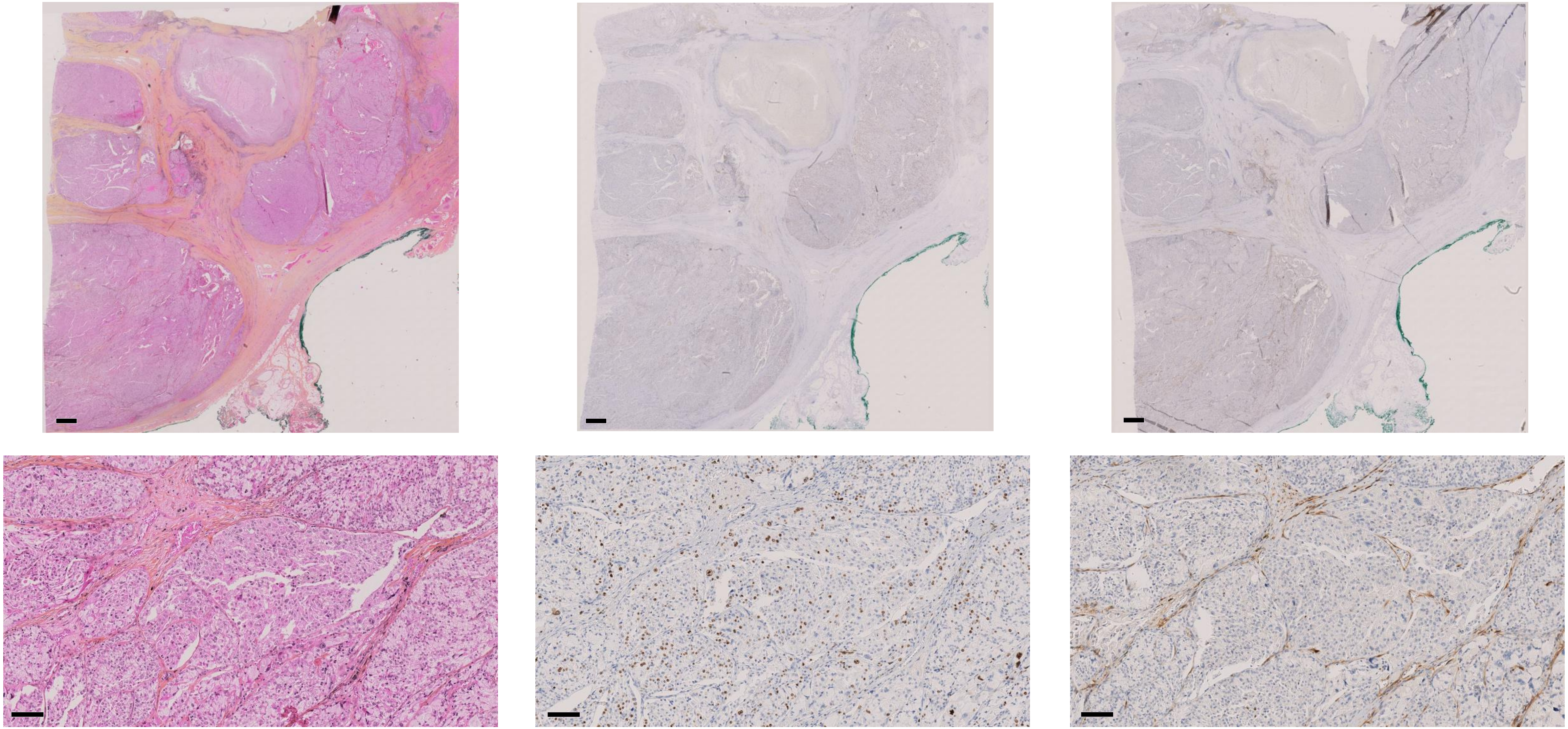

HE KI67 p16

Scale bar = 1mm
Scale bar = 100µm

PDTC19 - JPI40 - pCDK4 profile H

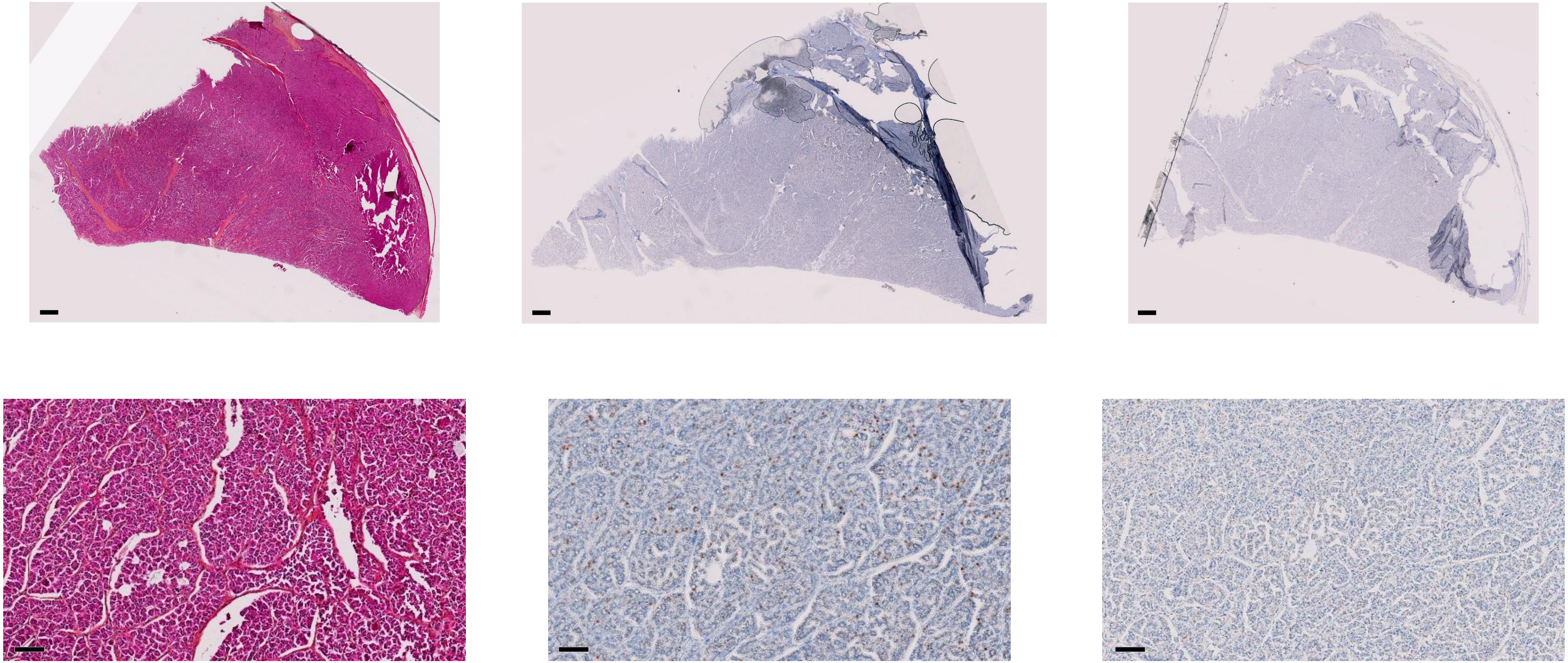

HE | KI67 | p16

Scale bar = 1mm
Scale bar = 100µm

PDTC15 - JPI68 - pCDK4 profile H

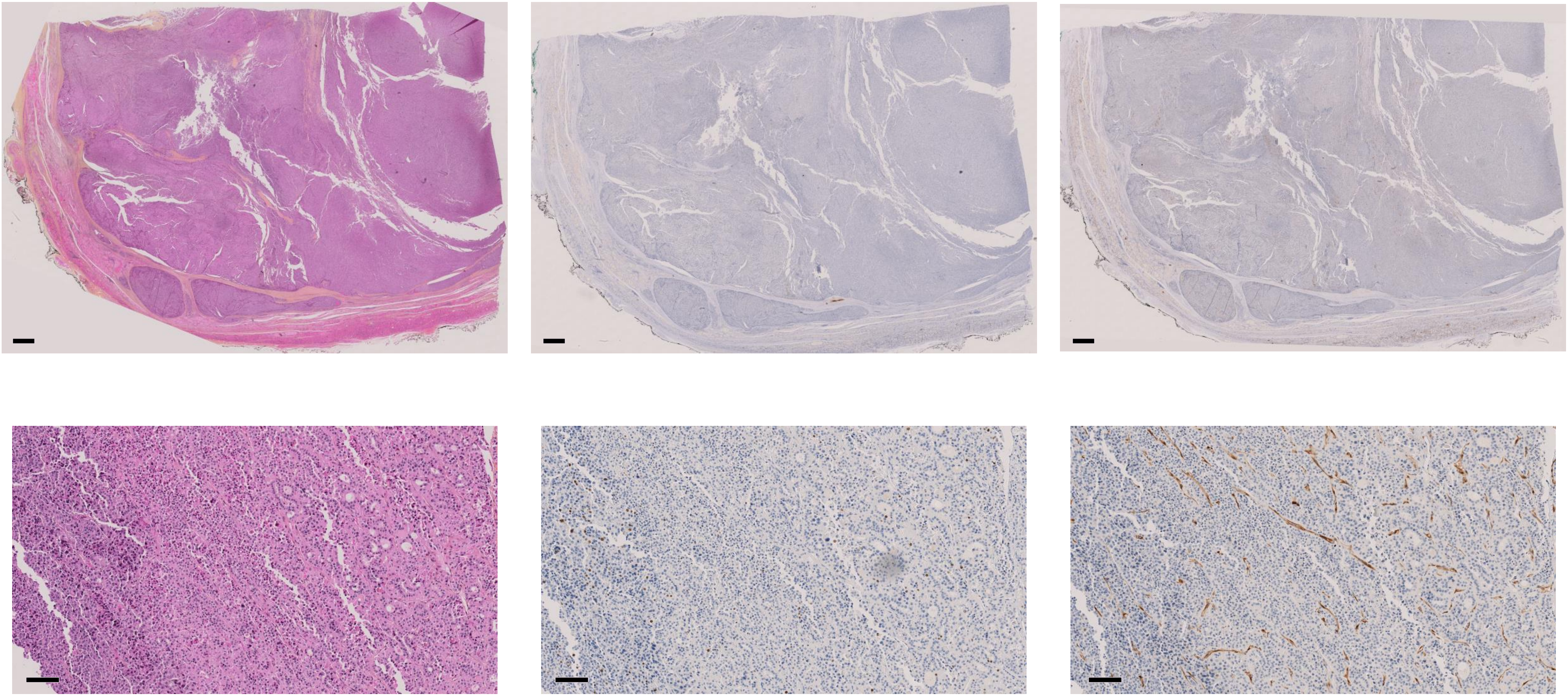

HE KI67 p16

Scale bar = 1mm
Scale bar = 100µm

PDTC3 - JPI83 - pCDK4 profile L

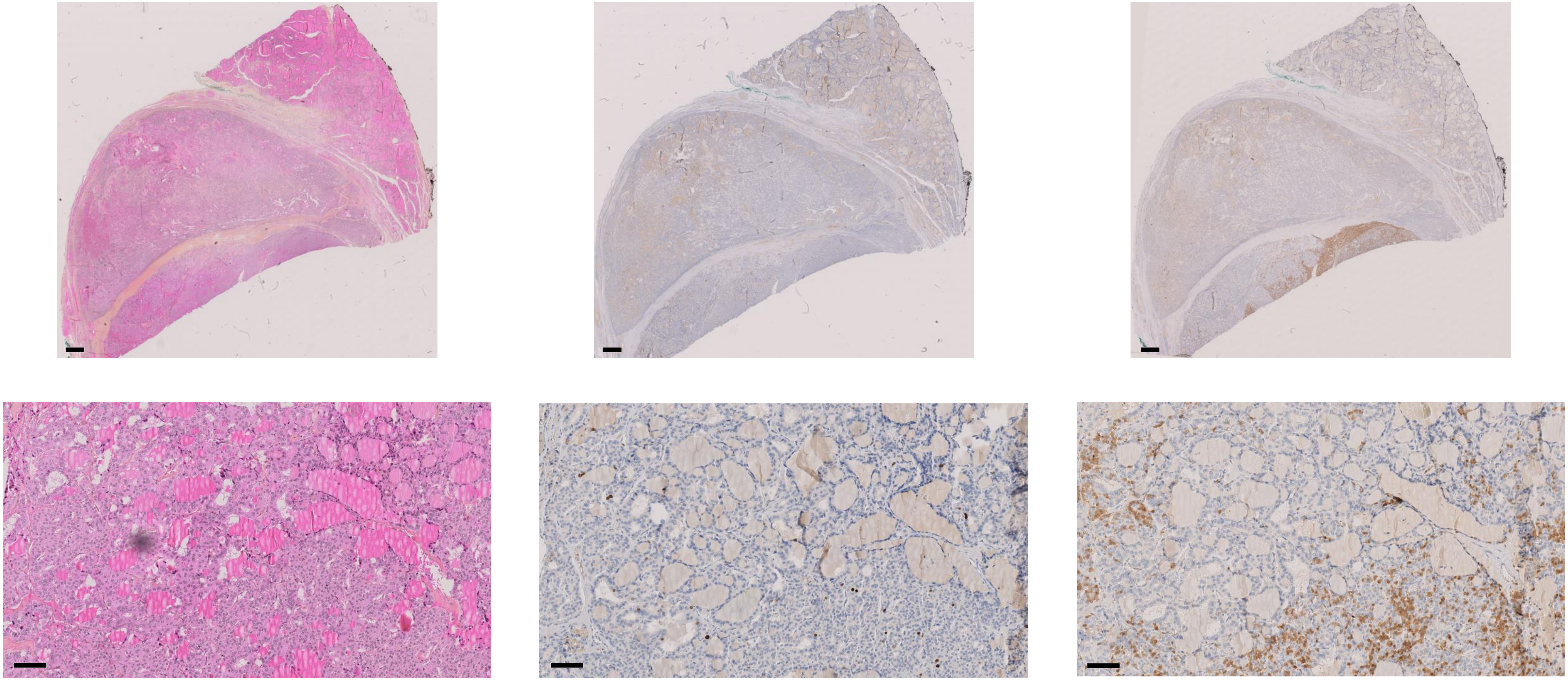

HE **KI67** **p16**

Scale bar = 1mm

Scale bar = 100µm

PDTC8 - JPI33 - pCDK4 profile L

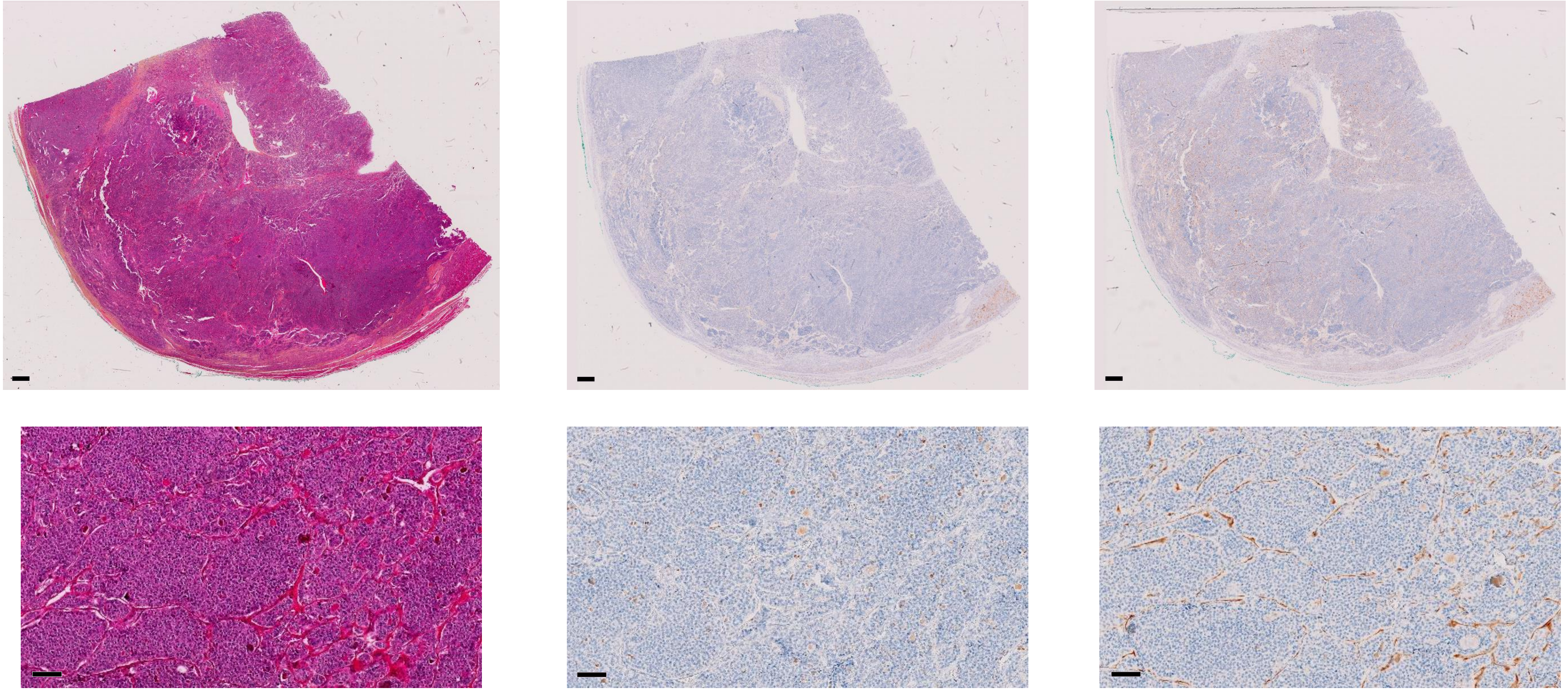

HE KI67 p16

Scale bar = 1mm

Scale bar = 100µm

PDTC7 - JPI31 - pCDK4 profile L

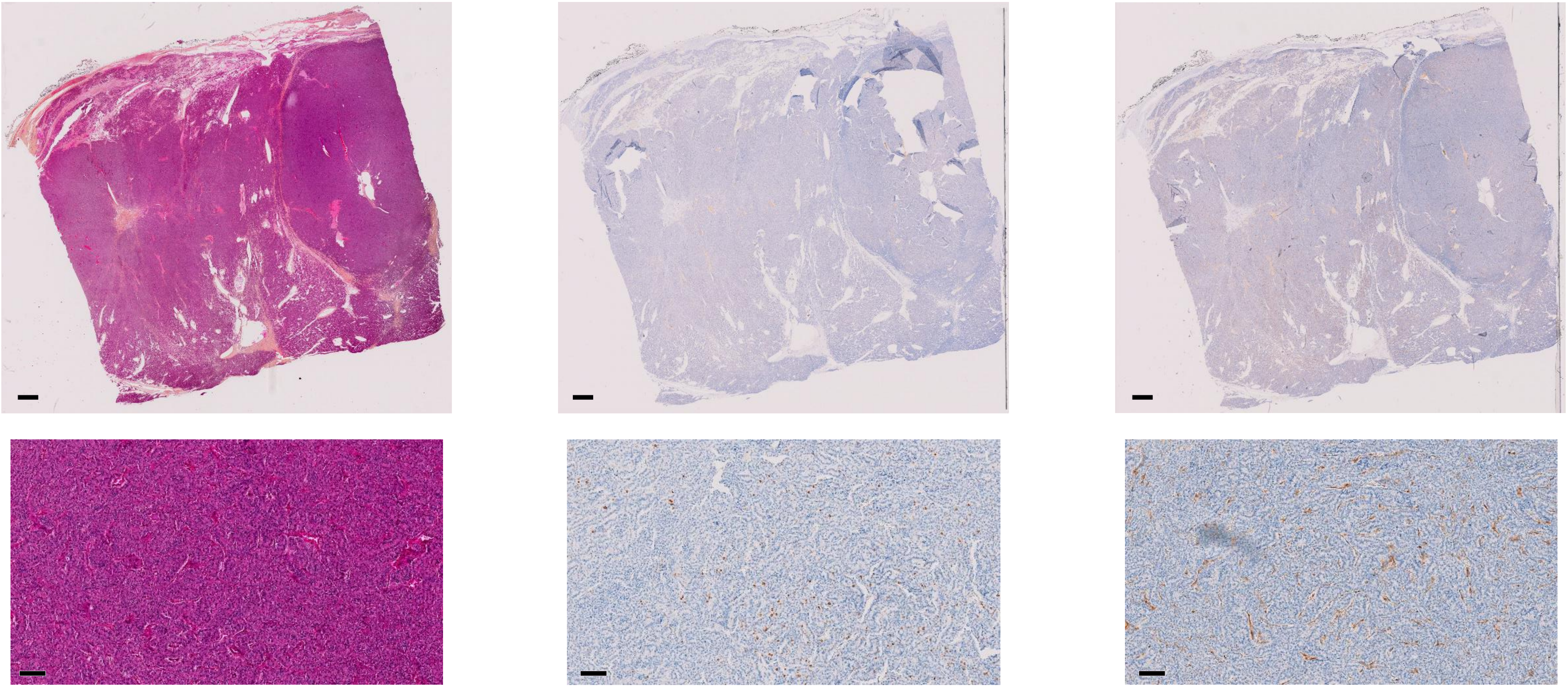

HE **KI67** **p16**

Scale bar = 1mm

Scale bar = 100µm

PDTC6 - JPI82 - pCDK4 profile L

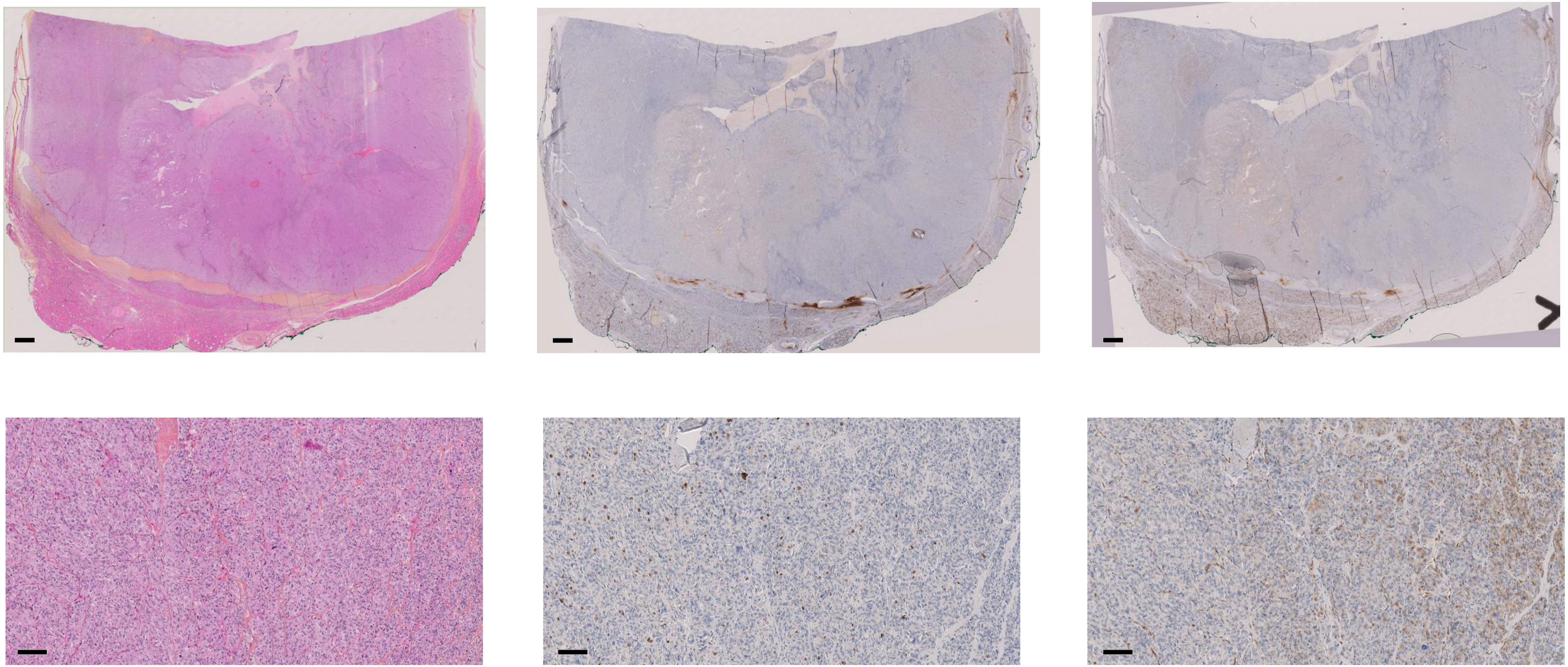

HE **KI67** **p16**

Scale bar = 1mm
Scale bar = 100µm

PDTC11 - JPI79 - pCDK4 profile L

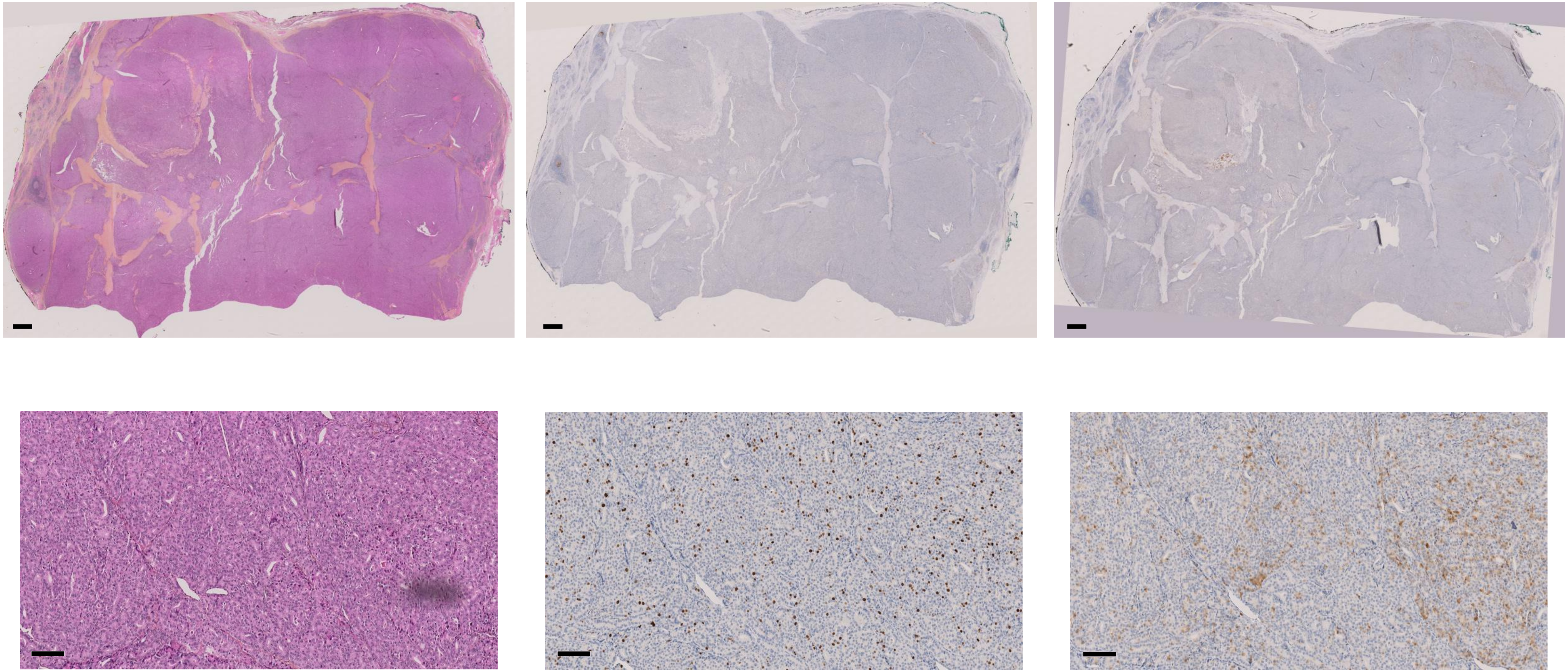

HE **KI67** **p16**

Scale bar = 1mm
Scale bar = 100µm

PDTC2 - JPI70 - pCDK4 profile L

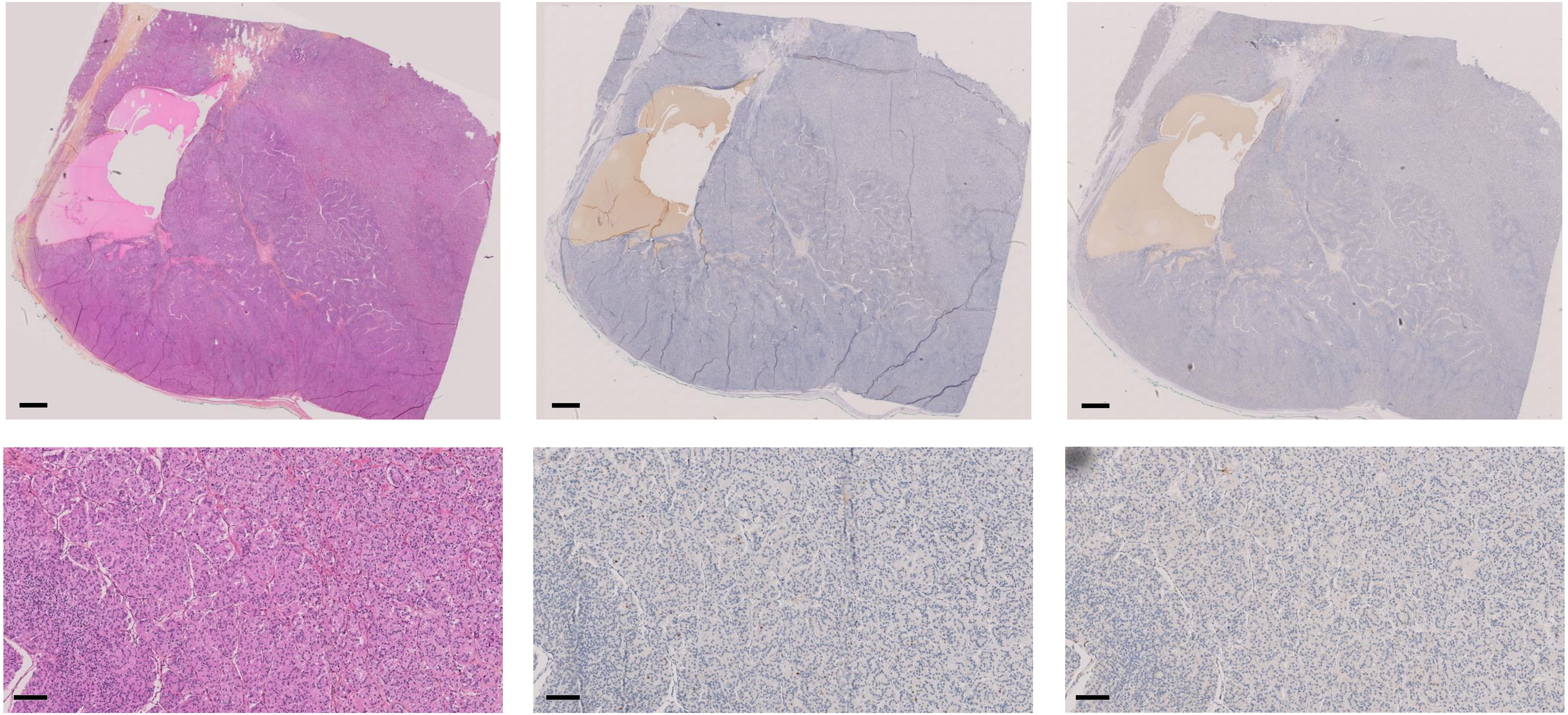

HE

KI67

p16

Scale bar = 1mm

Scale bar = 100µm

PDTC21 - JPI22 - pCDK4 profile with only spot 1

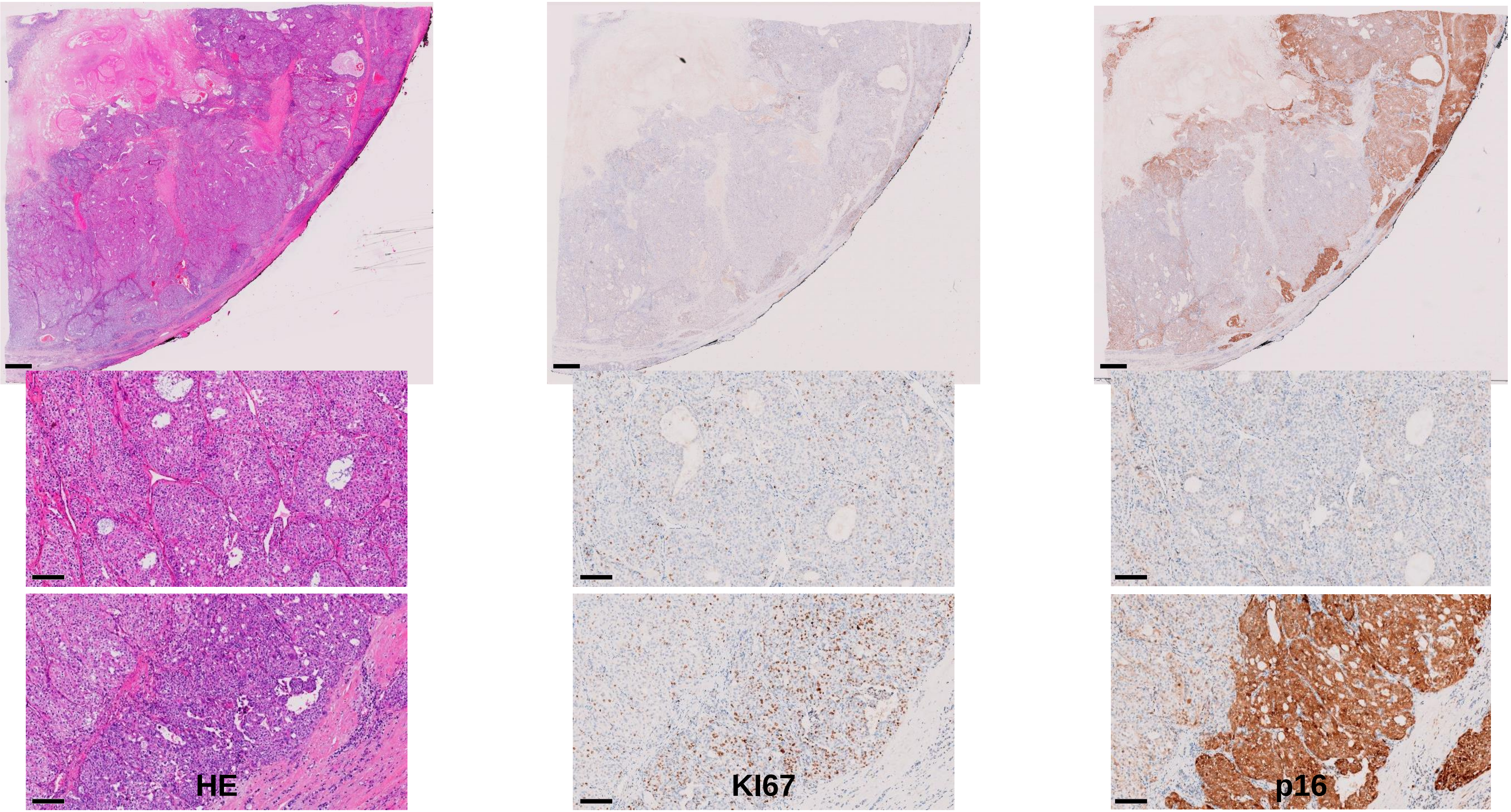

PDTC22 - JPI17 - pCDK4 profile H

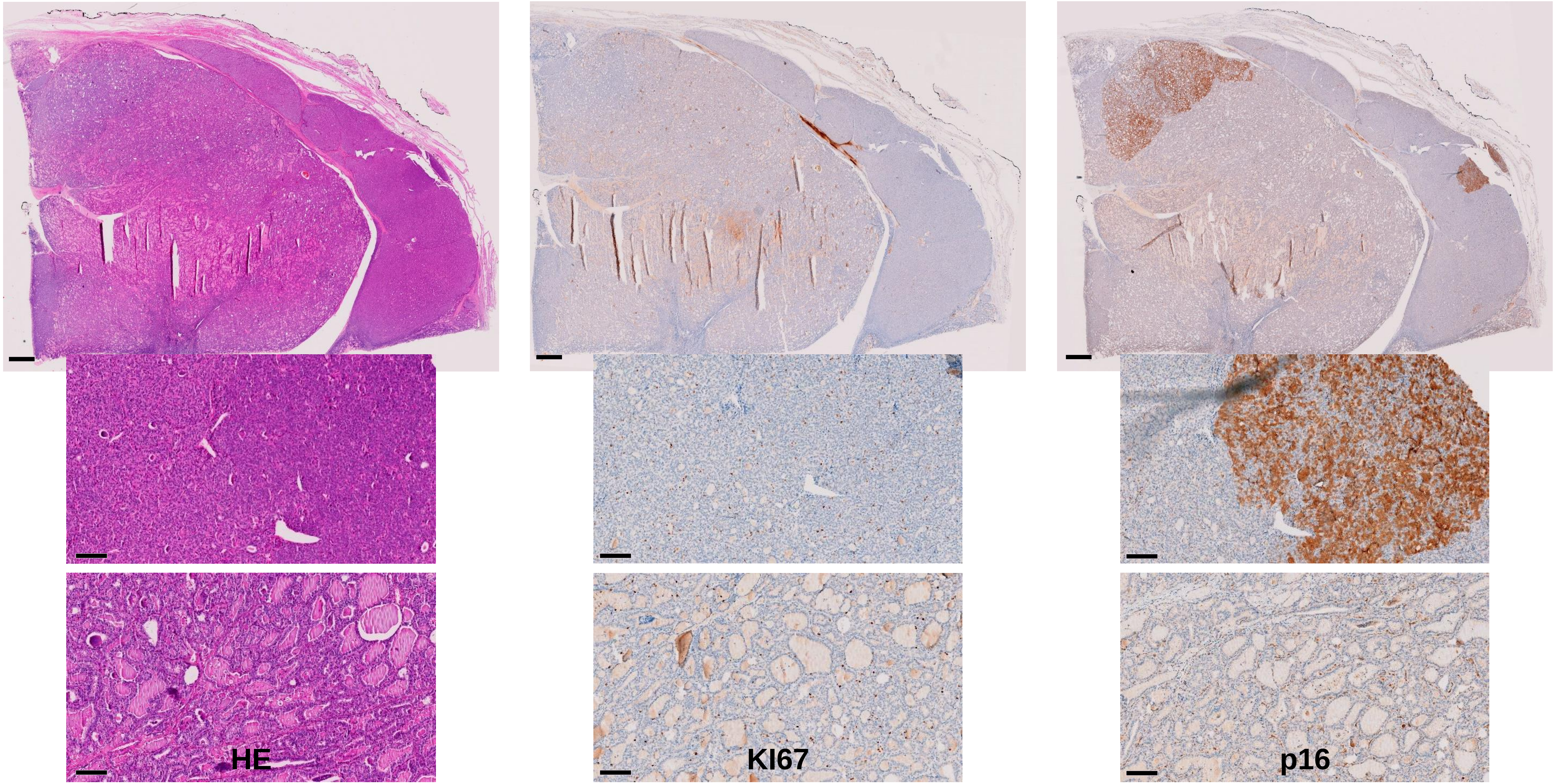

PTC17 - JPI24 - pCDK4 profile H

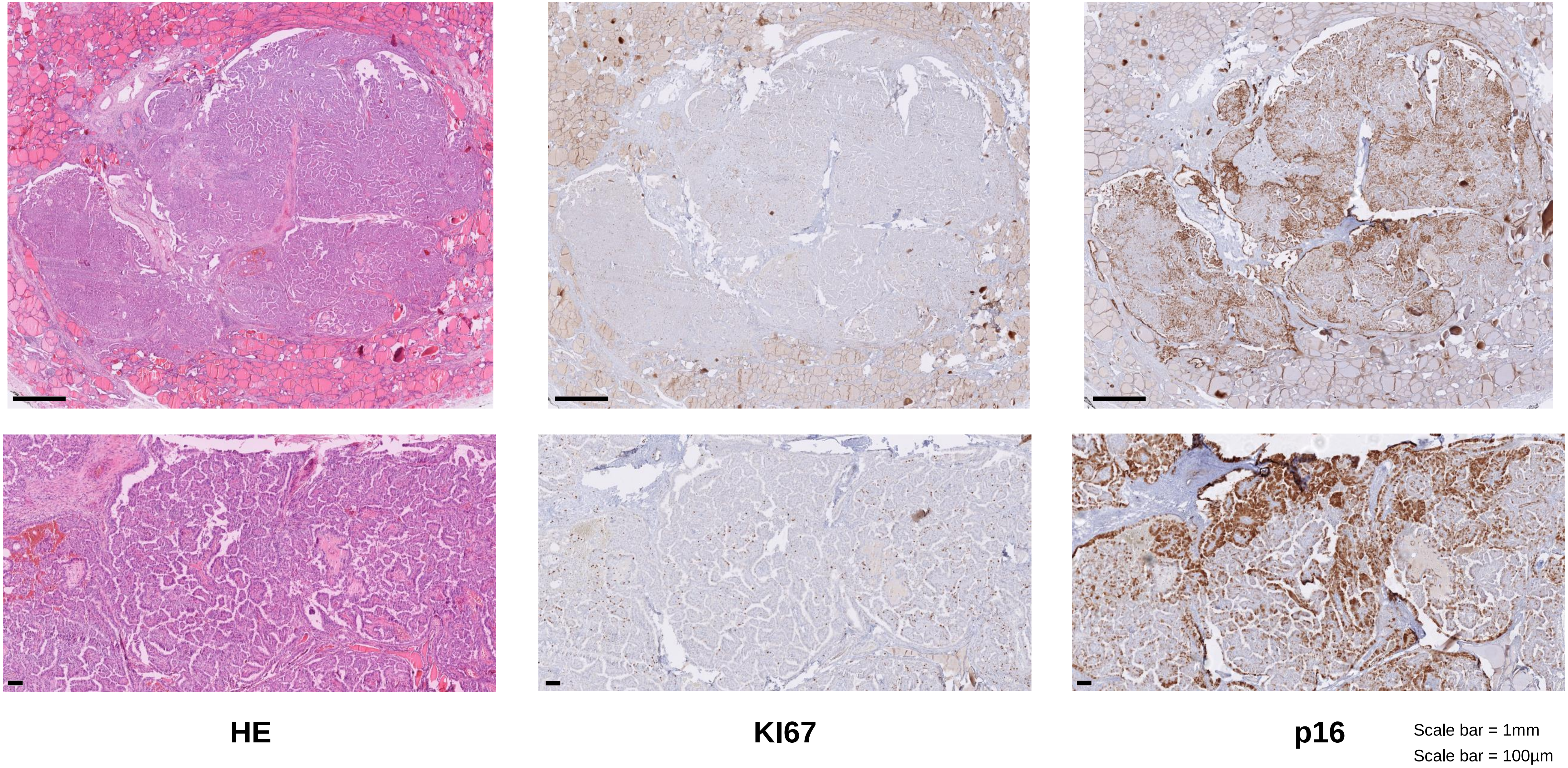

PTC13 - JPI36 - pCDK4 profile H

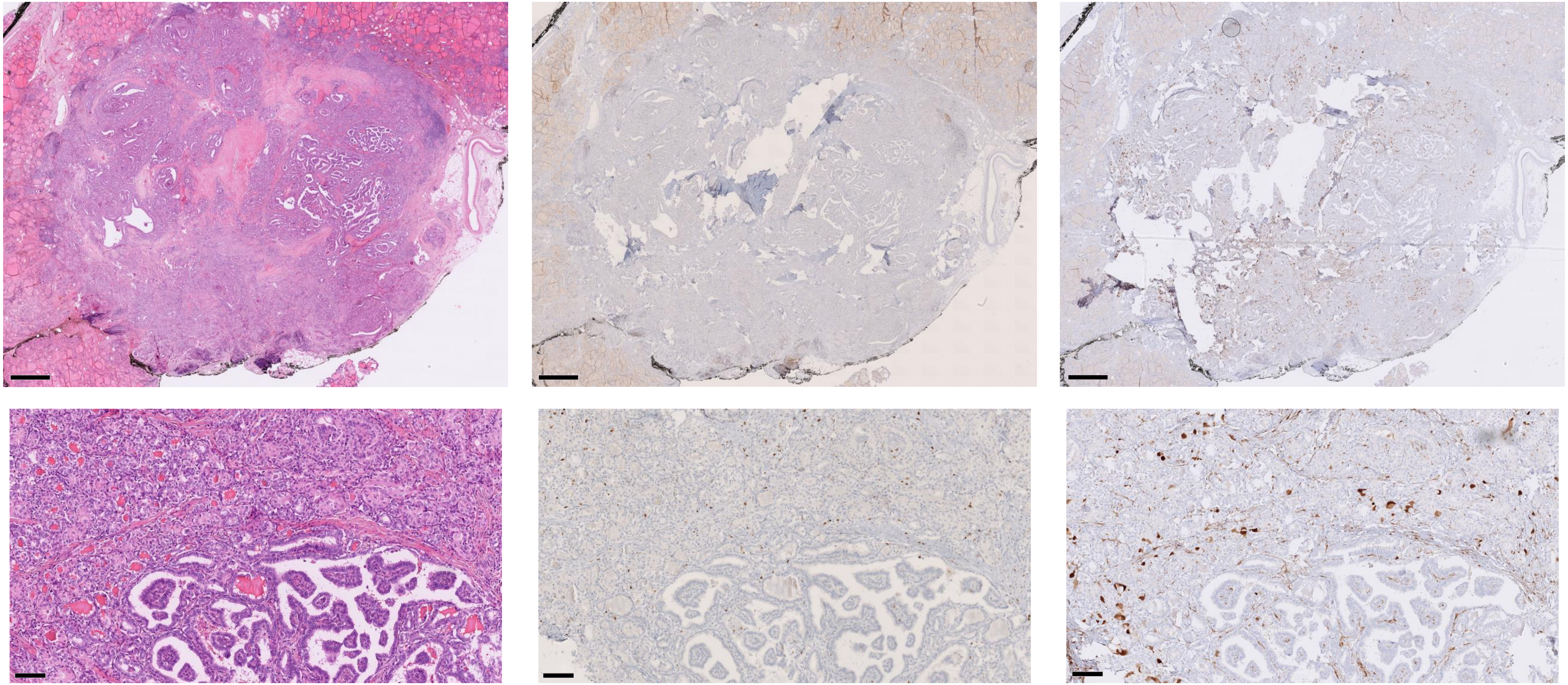

HE | KI67 | p16

Scale bar = 1mm
Scale bar = 100µm

PTC6 - JPI65 - pCDK4 profile L

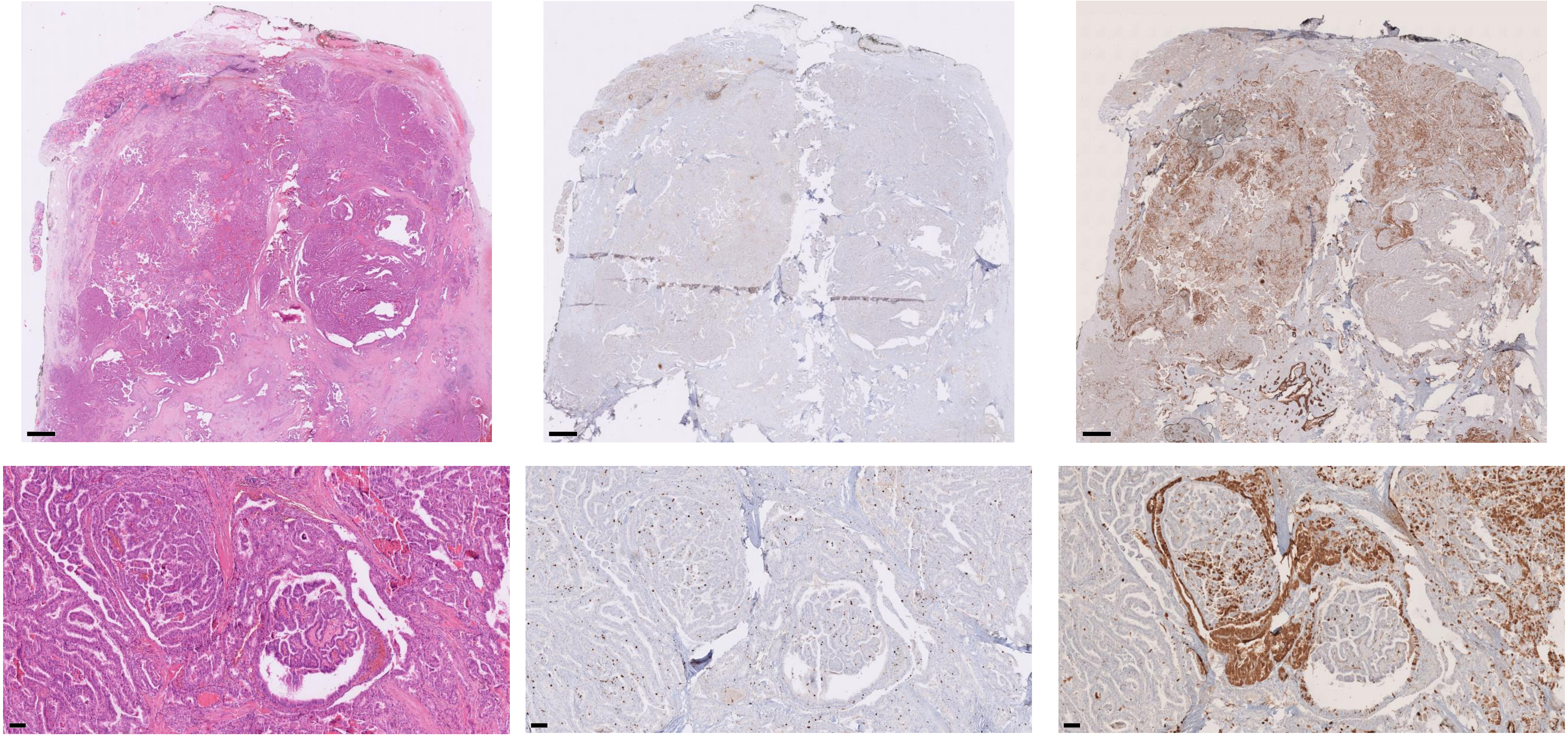

HE KI67 p16

Scale bar = 1mm
Scale bar = 100µm

PTC2 - JPI61 - pCDK4 profile L

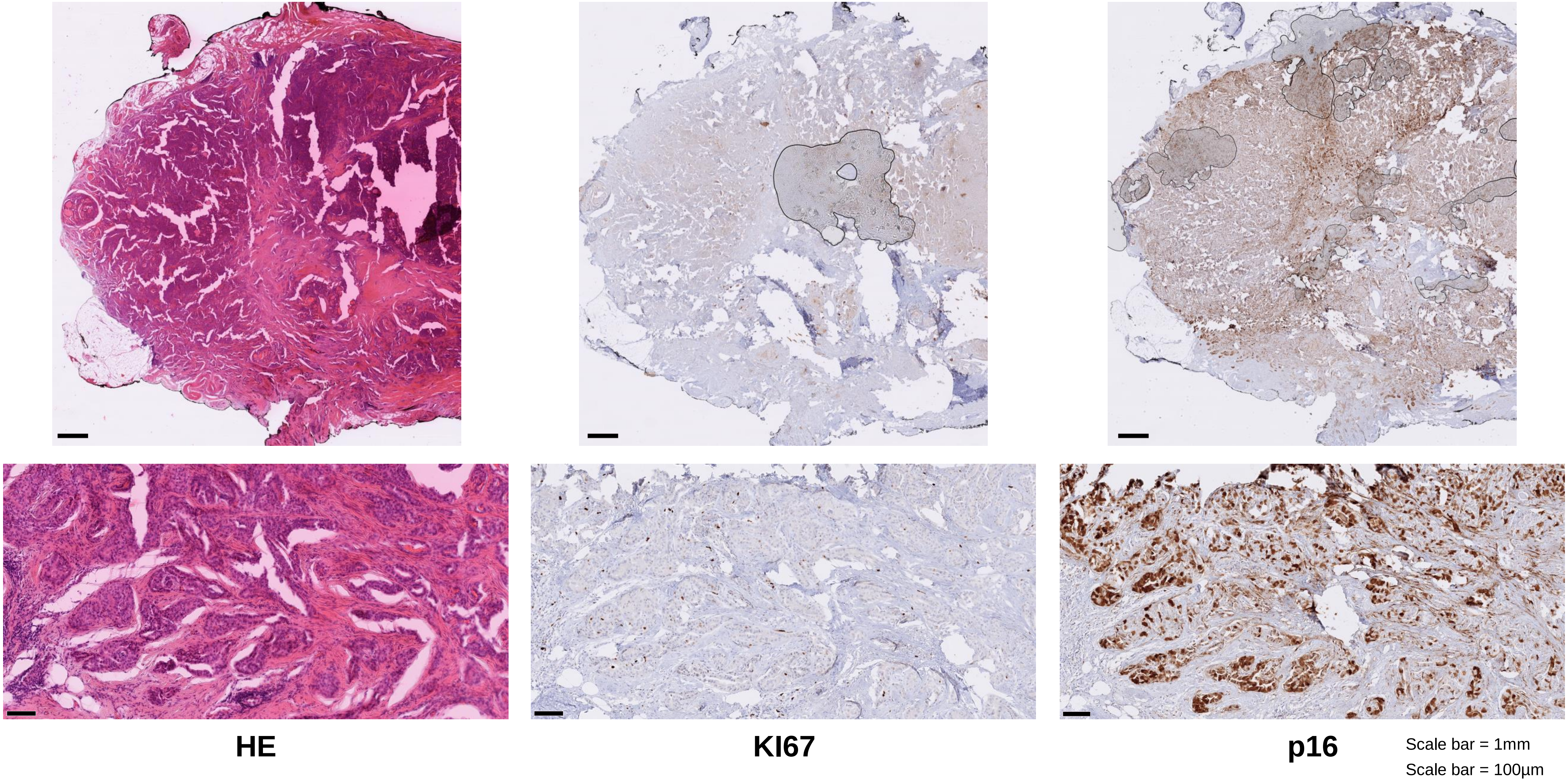

PTC11 - JPI64 - pCDK4 profile L

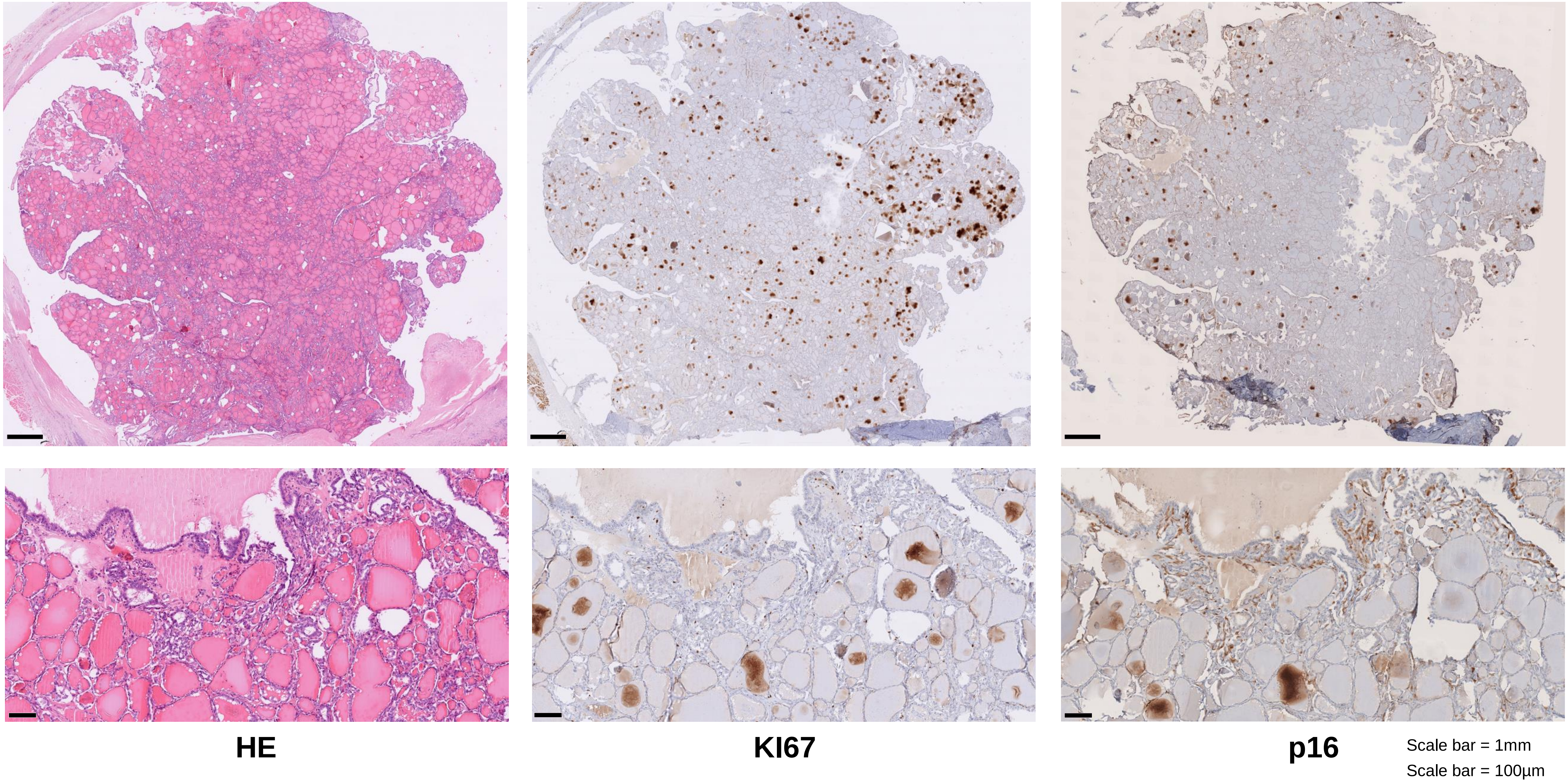

PTC5 - JPI58 - pCDK4 profile L

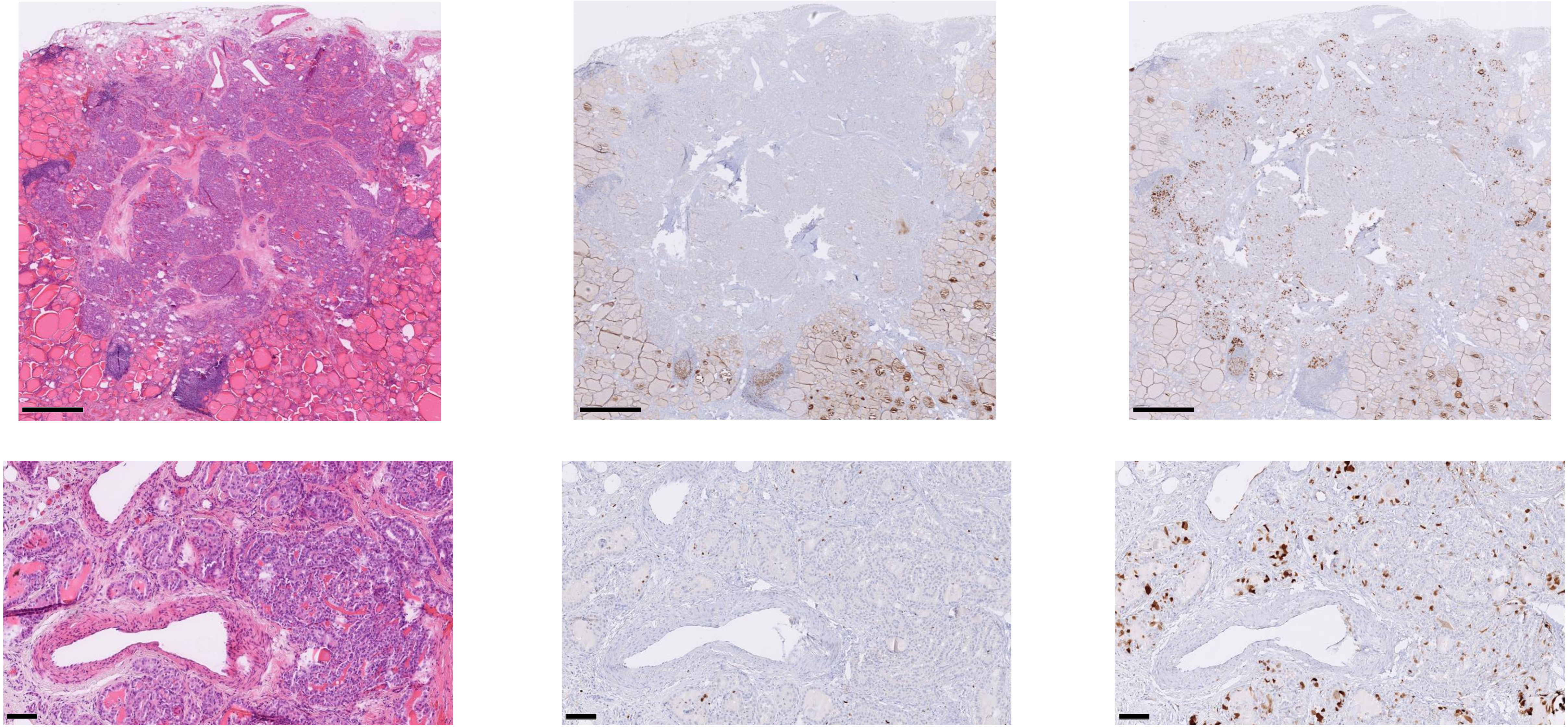

HE KI67 p16

Scale bar = 1mm
Scale bar = 100µm

PTC2N (normal tissue) - JPI56 - pCDK4 profile L

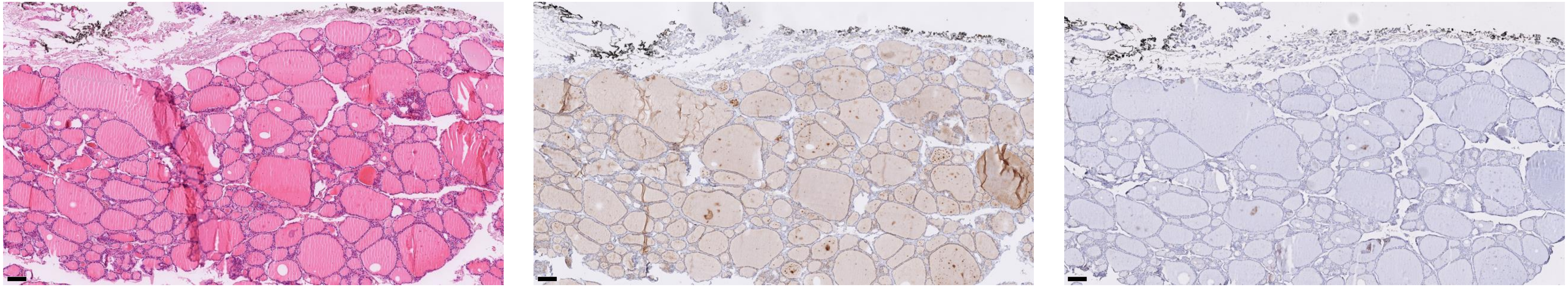

PTC11N (normal tissue) - JPI57 - pCDK4 profile A

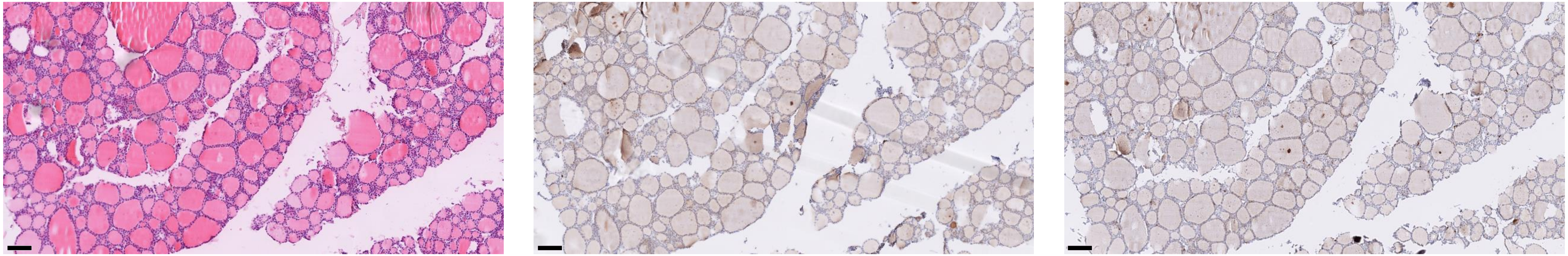

HE | KI67 | p16

Scale bar = 100µm
